# Effects of *Lactobacillus plantarum* P9 Probiotics on Defecation and Quality of Life of Individuals with Chronic Constipation: Protocol for a Randomized, Double-Blind, Placebo-Controlled Clinical Trial

**DOI:** 10.1155/2022/4144321

**Published:** 2022-06-13

**Authors:** Wenjun Liu, Nong-Hua Lu, Xu Zhou, Yingmeng Li, Yong Xie, Longjin Zheng, Weifeng Zhu, Qiuping Xiao, Ni Yang, Kexuan Zuo, Qingni Wu, Tielong Xu, Heping Zhang

**Affiliations:** ^1^Key Laboratory of Dairy Biotechnology and Engineering Ministry of Education, Key Laboratory of Dairy Products Processing Ministry of Agriculture and Rural Affairs, Inner Mongolia Key Laboratory of Dairy Biotechnology and Engineering, Inner Mongolia Agricultural University, Hohhot 010018, China; ^2^Department of Gastroenterology, The First Affiliated Hospital of Nanchang University, Nanchang 330006, China; ^3^Evidence Based Medicine Research Center, Jiangxi University of Chinese Medicine, Nanchang 330004, China; ^4^State Key Laboratory of Innovative Medicines and High-efficiency Energy-saving Pharmaceutical Equipment, Nanchang 330006, China

## Abstract

**Background:**

Although probiotics have been shown to improve constipation-related symptoms, a clear consensus on the use of probiotics as a constipation-relieving agent has not been reached, which is attributed to the limited available evidence and inconsistent protocols used in existing studies.

**Method:**

A randomized, double-blind, placebo-controlled clinical trial is designed to study the efficiency and possible mechanism of action of probiotics for chronic constipation, in which 200 eligible volunteers with chronic constipation will be randomly assigned to a probiotic group (oral *Lactobacillus plantarum* P9 probiotic powder, 100 billion colony-forming units (CFUs)/day) or a placebo group. Volunteers, treatment distributors, data collectors, and data analysts will be blinded. The primary outcome is the weekly mean frequency of complete spontaneous bowel movements (CSBMs), and secondary outcomes include weekly mean frequency of CSBMs ≥3, weekly mean frequency of spontaneous bowel movements (SBMs), weekly mean stool appearance score, weekly mean difficulty of passing stool score, weekly percentage of volunteers who use auxiliary measures to assist with defecation (WPUAMA), quality-of-life (QOL) score, emotional status score, gut microbiome, and faecal metabolome. Each outcome measure will be assessed at the time points of preadministration (day 0), administration (day 14 and/or 28), and postadministration (day 42) to identify inter- and intragroup differences. Adverse events will be recorded to evaluate the safety of *L. plantarum* P9. *Discussion*. The protocol will provide methodological guidance for other similar studies, avoiding methodological bias and ultimately facilitating the formulation of consensus on the use of probiotics as a constipation-relieving agent. In addition, the results are more comprehensive than those of existing studies and may objectively and scientifically reflect the effectiveness of *L. plantarum* P9 on constipation. If the expected study findings are obtained, *L. plantarum* P9, taken as a probiotic, may become a complementary choice for chronically constipated patients. This trial is registered with Chinese Clinical Trial Registry (ChiCTR) (no. ChiCTR2000038396) registered on November 22, 2020, https://www.chictr.org.cn/showproj.aspx?proj=54024.

## 1. Background

Constipation is a common diagnosis made by gastroenterologists based on the assessment of infrequent bowel movements (<3 per week) and difficult stool passage, while patients may report multiple symptoms, including a sense of incomplete defecation, abdominal pain, bloating, excessive straining accompanied by a sensation of anorectal blockage during stool passage, and requiring manual assistance to release the stool. Acute (or nonchronic) constipation either results in blockage of the intestinal tract that requires surgery [1] or tends to be ignored by patients. Academically, constipation always refers to chronic constipation, which is categorized into primary constipation and secondary constipation (attributed to other diseases or factors). Based on the Rome IV criteria [2], the classifications of primary constipation are functional constipation, irritable bowel syndrome with constipation, and defecatory disorders. From the aspect of the transiting rate of stool movement through colonic and contraction of muscle tissue during defecation, primary constipation is also divided into slow transit constipation, normal transit constipation, and defecatory disorders [3].

The global prevalence of constipation ranges from 10% to 30% [4–7]. The variance in prevalence may be attributed to the individuals assessed in different surveys, which may be self-reported or use different Rome criteria (I, II, III or IV) to identify participants [5, 8–10], thus lacking a concise definition of constipation. Overall, chronic constipation is more frequent in the population with the following characteristics: elderly, female, nonwhite race, medication intake, low income and education level, physical inactivity, and depression [8, 11–23]. Because patients are constantly suffering from physical symptoms and psychological distress, chronic constipation potentially disturbs people's lives, studies, and work [24, 25] due to dyspareunia, sexual dysfunction, urine retention [26], reduced mental health and social function [24, 27], school absenteeism, a high number of lost work days, and the cost of medical care [28].

Only one-fifth of patients with constipation seek medical advice [19], and laxatives are the most frequently prescribed agents [29, 30]. Laxatives, physical exercise, fibre intake, and dietary management are the traditional treatments. However, approximately 50% of constipated individuals are dissatisfied with their current treatments [31, 32]. As many as 74% of nursing home residents have been reported to use laxatives daily [33–35], of which only 28%–57% are estimated to be satisfied with the treatment according to the results of two Internet-based surveys [36, 37]. Undesirable side effects are a main reason for suboptimal satisfaction. New treatments must be developed for unsatisfied patients, and daily intake of probiotics holds great promise since various probiotics have shown benefits for constipation treatment [38–43].

However, although many studies have reported that probiotics are potentially beneficial for constipated individuals [39–43], a clear consensus on the use of probiotics as a constipation-relieving agent has not been reached [44]. This lack of consensus is mainly attributed to the shortage of study methodology in terms of incorrect statistical analysis, inconsistent definitions of constipation and outcomes of the intervention, and an unvalidated assessment technique [44–46]. Therefore, high-quality randomized controlled trials (RCTs) are needed to reach a more confident consensus [47]. Moreover, RCTs examining the effectiveness of probiotics as a constipation-relieving agent may elucidate a clear mechanism of action for a more confident conclusion. Generally, a balanced intestinal flora provides health benefits to human hosts; conversely, an imbalanced flora may promote the development of constipation [48]. Consistently, probiotics may positively modify the intestinal flora [49, 50] and even provide potentially beneficial microorganisms to the host.

Thus, we designed this protocol to study the effectiveness of *L. plantarum* P9 on chronic constipation and to guide other studies assessing the effectiveness of probiotics. The correlation between the clinical improvement of symptoms and changes in intestinal flora was also analysed.

## 2. Methods and Design

### 2.1. Design

**Figure 1:**
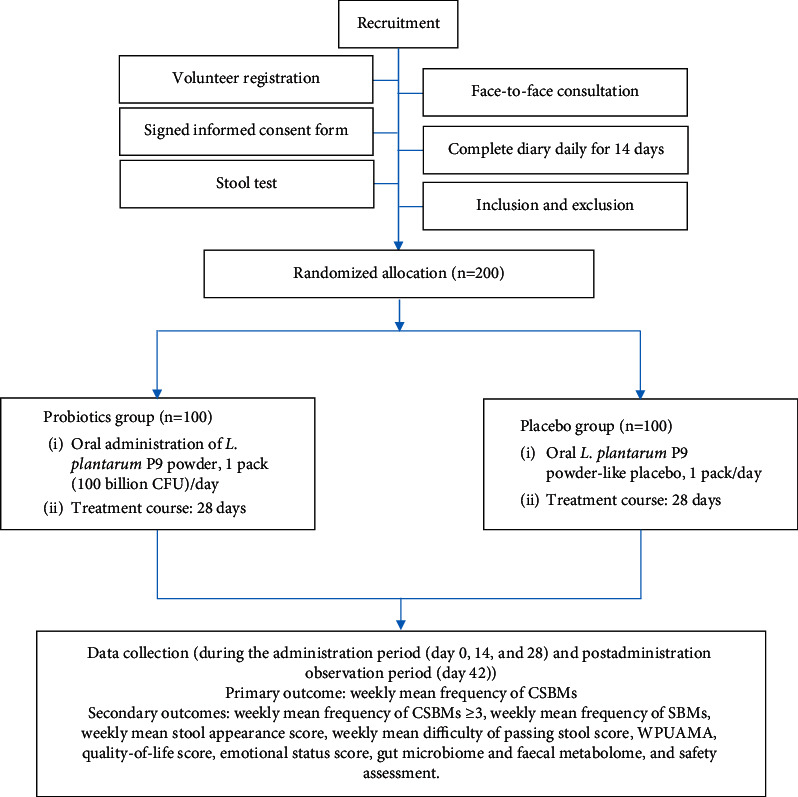
Flowchart of the protocol.

This study is a randomized, double-blind, placebo-controlled clinical trial conducted in Nanchang, China, and volunteers may receive the intervention (oral probiotics or placebo) in their own homes. The protocol was prospectively registered at the Chinese Clinical Trial Registry (ChiCTR) (NO. ChiCTR2000038396) (Appendix 1) and approved by the Ethics Committee of the First Affiliated Hospital of Nanchang University (Approval Number: IIT [2020], Clinical Ethics Review NO. 004) (Appendix 2). This manuscript is prepared according to the Standard Protocol Items: Recommendations for Interventional Trials (SPIRIT) [51]. The flowchart of the trial process is provided in Figure 1.

### 2.2. Inclusion Criteria

Eligible patients should fully meet the Rome IV criteria [2]:

(1) Onset of the following symptoms for at least 6 months before enrolment and symptoms within the past 3 months that meet the following criteria [2, 52]:
  1. Two or more of the following symptoms: (a.) difficulty passing stool, at least 25% of defecations; (b.) lumpy or hard stool, at least 25% of defecations (Bristol Stool Form Scale (BSFS) types 1 or 2 (Appendix 3) [53]); (c.) incomplete defecation in at least 25% of defecations; (d.) sense of anorectal obstruction in at least 25% of defecations; (e.) need for manual assistance for defecation (such as using fingers to assist with defecation or pelvic floor support) in at least 25% of defecations; and (f.) fewer than 3 spontaneous bowel movements (SBMs) per week.
  2. Loose stool rarely occurs without the use of laxatives.
  3. Insufficient stools are rarely present without the use of laxatives.
(2) Willing to sign the informed consent form (Appendix 4)
(3) The volunteers involved in this study will be patients with chronic constipation aged 18–65 years. For patients aged from 18 (exclusive) to 50 (inclusive) years, the result of stool tests (including occult blood) conducted during the screening period must be normal or abnormal but determined to be clinically irrelevant by the investigators. For patients aged 50 (exclusive) to 65 (inclusive) years, the result of a colonoscopy performed at a tertiary or higher-level hospital within the past 6 months must be normal or abnormal but determined clinically irrelevant by the investigators.

### 2.3. Exclusion Criteria

Volunteers with any of the following conditions will be excluded:

1. Personal or family history of colon cancer, celiac disease, or inflammatory bowel disease
2. Intestinal organic diseases confirmed on a previous colonoscopy
3. Plans to become pregnant or father a child in the next 3 months, or pregnant, or breastfeeding
4. Allergies to samples or ingredients
5. Use of antibiotics or probiotics within the past two weeks
6. Use of antianxiety, antidepressant, or other psychotropic drugs within the past month
7. Need for long-term use of medications for constipation
8. History of severe diseases, such as myocardial infarction, cerebral infarction, and malignant tumour, judged by the investigators as disqualifying conditions
9. Major mental illnesses, inability to control one's actions, or inability to cooperate
10. Illiteracy, inability to understand the informed consent form, or inability to independently sign the informed consent form

### 2.4. Volunteer Recruitment

**Figure 2:**
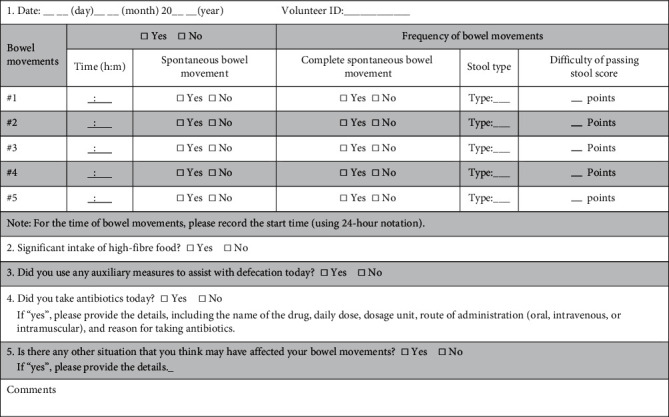
Defecation diary.

The investigators will recruit volunteers from the public through in-person communication, posters, and WeChat promotions. Volunteers can scan a WeChat two-dimensional (QR) code to register for enrolment, submit their personal information, and answer questions related to inclusion and exclusion criteria. Volunteers will undergo a procedure that includes three rounds of screening. First, the investigators will collect and collate the volunteers' registration information and, according to the information provided by registrants, invite potentially eligible candidates to participate in a central consultation with clinical specialists for further screening and selection of eligible volunteers. Second, during the central consultation, trained study implementers will introduce the trial to volunteers in the form of a video and information sheets describing the main aspects of the trial and discuss the information provided in the video and information sheets. Then, volunteers will meet with the clinical specialist to confirm their registration information, especially regarding inclusion and exclusion criteria. Third, volunteers will sign an informed consent form in the presence of a member of the data management team (DMT). Then, volunteers will undergo a 14-day screening period when they will not be allowed to take any medicines or health products to improve their constipation symptoms. Each volunteer will be asked to collect one stool sample and complete an online diary entry daily (Figure 2). At the end of the screening period, the diary and stool sample results will be reviewed and used to select eligible volunteers. Subsequent steps will include the formal intervention and follow-up visits (Figure 1).

### 2.5. Randomization and Blinding

Eligible volunteers will be assigned a unique serial number (e.g., 001, 002, 003, 004, 005……). The unique number will be used as the volunteers' ID throughout the study period to guarantee anonymity and confidentiality. For each of these unique numbers, a random sequence will be generated by the computer software *R* 4.1.0 and used to randomly assign the unique number (participant) to the probiotic group or the placebo group. During the study, the volunteer's treatment distributors, data collectors, and data analysts will be blinded to the randomization sequence. The randomization sequence will be maintained by an independent project administrator and will only be unblinded in the case of major safety issues or when performing the interim and final data analyses. Moreover, an independent project administrator will label the probiotics or placebo packages with unique numbers corresponding to random sequences in advance to achieve allocation concealment. During the period of administration, the distributor will distribute the treatment packages according to the unique number that corresponds to each participant.

### 2.6. Study Interventions

The *L. plantarum* P9 and placebo powders will be manufactured in parallel and independently by Research and Development Department, Jiangzhong Pharmaceutical Company Limited (Nanchang, China). Briefly, the preparation procedure consists of three steps: weighing, mixing, and packaging. *L. plantarum* P9 powder is composed of 20% maltodextrin, 20% orange powder, 20% maltitol, and 40% *L. plantarum* P9, while placebo powder consists of 60% maltodextrin, 20% orange powder, and 20% maltitol. Raw materials for *L. plantarum* P9 and placebo powders are mixed using a Hopper mixer (hit-400) and sealed into 2 g packages. The temperature is controlled at 25 ± 2°C, and the relative humidity is less than 65% during the three steps. In the study, when the product of *L. plantarum* P9 powder is manufactured, a randomized sample is sent to Jinhua Yinhe Biotechnology Co., Ltd. (https://yinhewdy168.foodmate.net/) for quality inspection. The activity of probiotics is greater than 67 billion colony-forming units (CFUs)/g.

The study will include three phases (Table 1), including a screening period (an observation period prior to treatment administration), an observation period during administration, and an observation period after administration. The interventions in each phase will be administered as follows:

1. Period of screening (preadministration observation period) (days 14 to 0): in this phase, volunteers will not receive the intended interventions, i.e., probiotics and placebo. Eligibility screening will be performed as described during volunteer recruitment (Table 1).
2. Observation period during administration (days 0 to 28): (1) Probiotics group: volunteers will take 1 package of *L. plantarum* P9 powder directly or with warm water (below 40°C) on a full stomach at a dose of 100 billion CFUs per day; if antibiotics must be taken, probiotics should be taken 2 hours later. (2) Placebo group: volunteers will take the placebo in the same manner as the probiotics group. The placebo contains no probiotics but has the same appearance, packaging, and taste as the *L. plantarum* P9 powder.
3. All remaining probiotics or placebo (unused) and empty (used) packages will be collected at the end of this period to monitor compliance. During the study, both probiotics and placebo will be stored in a cool, dry place away from direct sunlight.
4. Postadministration observation period (days 29 to 42): no probiotics or placebo will be taken.

### 2.7. Prohibited Confounding Interventions

The following treatments are prohibited during the study: (1) probiotics, prebiotics, and foods containing probiotics (such as yoghurt) other than the *Lactobacillus plantarum* used in this study; (2) antianxiety, antidepressants, and other psychotropic drugs; and (3) other substances designed to improve intestinal symptoms.

In addition, antibiotics will be monitored during the study, and normal dietary habits will be recommended. All concomitantly used substances/drugs related to defecation should be recorded daily in the online defecation diary (Figure 2), and explanations are needed.

### 2.8. Primary Outcome

A defecation diary developed with reference to the literature [32, 53–56] will be completed daily online by the participants (Figure 2). Based on the diary, the changes in primary and some of the secondary outcomes from day 0 (week 0) to day 42 (week 6) will be evaluated.

1. The primary outcome measure is the weekly mean frequency of complete spontaneous bowel movements (CSBMs) [32, 54, 55].

A CSBM is defined as the ability to achieve a complete bowel movement without the use of any drugs or other auxiliary measures during the previous 24 hours. Based on the diary (Figure 2), the weekly mean frequency of CSBMs from day 0 (week 0) to day 14 (week 2), day 28 (week 4), and day 42 (week 6) will be evaluated [32, 54, 55] (Table 1).

### 2.9. Secondary Outcomes

1. Based on the diary (Figure 2), the changes in the percentage of volunteers with a weekly mean frequency of CSBMs ≥3 [32, 54, 55], changes in the weekly mean frequency of spontaneous bowl movements (SBMs) [32, 54–56], weekly mean stool appearance score [53], weekly mean difficulty of passing stool score, and weekly percentage of volunteers who use auxiliary measures to assist with defecation (WPUAMA) will be evaluated each week from day 0 (week 0) to day 14 (week 2), day 28 (week 4), and day 42 (week 6). An SBM is defined as a bowel movement achieved without the use of any drugs or other auxiliary measures during the previous 24 hours [32, 54, 55] (Table 1).
2. The Patient Assessment of Constipation Quality-of-Life (PAC-QOL) questionnaire (Appendix 5 [56, 57]) will be used to evaluate the QOL score on days 0, 14, 28, and 42.
3. The Depression, Anxiety and Stress Questionnaire (DASS-21) [58, 59] (Appendix 6) will be completed online on days 0 (baseline), 14, 28, and 42 and used to assess the participants' emotional status (depression, anxiety, and stress) within the past week (Table 1).
4. For the indicator of the gut microbiome, DNA will be extracted from the stool samples with the QIAamp Fast DNA Stool Mini Kit (Qiagen, Hilden, Germany) according to the manufacturer's instructions, and the quality of DNA will be examined using agarose gel electrophoresis and a NanoDrop spectrophotometer. Shotgun metagenomic sequencing will be performed on all samples using an Illumina HiSeq 2500 instrument. Libraries will be constructed from DNA fragments with a length of ∼300 bp; paired-end reads will be generated by sequencing 150 bp in the forward and reverse directions. Meanwhile, the metagenomic analysis will include several parts: the analysis of alpha diversity and beta diversity in each group to understand whether the differences in the microbiota compositions of groups are significant and a comparison of the taxonomic characteristics of the study group and the control group at the level of phylum, genus, and species to identify specific genes related to the individual differences of constipation. Metagenomic biological pathway analysis will be used to evaluate the effect of probiotics on the function of gut metagenomics in patients with constipation and to explore the metagenomic biological pathways contributing to the mechanism underlying the effect of probiotics on the treatment of constipation.
5. For the indicator of the faecal metabolome, stool samples will be extracted using the protein precipitation method, and the supernatant will be transferred to sample vials for LC-MS/MS analysis. The original data will be subjected to peak alignment, retention time correction, and peak area extraction using the XCMS-Plus program. The structure of metabolites will be identified by accurate mass matching (<5 ppm) and two-level spectrum matching, and the METLIN database will be retrieved. We will then delete data with missing values > 50% in the group, normalize the data, and conduct multidimensional statistical analysis, including unsupervised principal component analysis (PCA), supervised partial least squares discriminant analysis (PLS-DA), and potential differentially abundant metabolite analysis. The metabolomic analysis might further identify the potential differentially abundant metabolites of probiotics in the treatment of constipation, and a correlation analysis between the gut microbiota and metabolites will be performed.

### 2.10. Safety Evaluation

All related clinical trials have reported that probiotics are safe and do not induce significant adverse events compared with the placebo group. However, four types of adverse reactions related to probiotics should be considered, including symptoms that may be attributed to systemic infections, deleterious metabolic activities, excessive immune stimulation, and gastrointestinal side effects [60], which will be collected as possible adverse events during the study. Severe adverse events are those adverse events leading to study withdrawal, e.g., hospitalization, disability, mortal danger, or death. All adverse events, including symptoms, time of onset, duration, causal relationship to interventions, and measures taken, will be accurately recorded during the study. Any serious adverse events will be recorded along with the corresponding criteria and emergency measures and will be promptly reported to the Medical Ethics Committee of the First Affiliated Hospital of Nanchang University within 24 hours; a separate study report will be prepared. Based on the adverse events observed during the study, the safety of the probiotics will be evaluated as excellent (safe, without any adverse events), good (relatively safe with moderate adverse events that resolve on their own without any specific treatment and do not result in study withdrawal), conditional (adverse events that resolve after certain measures are taken and allow continued participation in the study), or unsafe (adverse events that result in study withdrawal) [61].

### 2.11. Additional Assessment

Demographic profiles, special situations related to defecation during the study, including changes in dietary habits (e.g., eating spicy or oily foods or drinking), taking antibiotics, and any other reported situation will be recorded and assessed as needed.

### 2.12. Sample Size

This study is a clinical trial during which volunteers will consume a sample medication or placebo for 28 days and will be followed up for 42 days. The primary outcome measure is the weekly frequency of CSBMs. The expected weekly frequencies of CSBMs on day 28 are 1.1 in the control group and 4.1 in the probiotic group, with a between-group difference of 3. Given a standard deviation *S* = 6, *α* = 0.05, and *β* = 0.20, the sample size should be at least 63 volunteers per group, as calculated with the formula for statistically significant effectiveness. After considering the drop-out rate (20% or lower), the sample size should be 76 or more participants per group. The final sample size will be 100 participants per group for a total of 200 volunteers.

### 2.13. Compliance Monitoring and Withdrawal

The probiotics and placebo will be supplied as packages distributed in boxes. At the start of the administration period, the appropriate number of probiotic and placebo packages will be distributed to the participants. All used (empty) and unused packaging will be kept by volunteers. At the end of the administration period, these packages will be collected to verify the number of remaining packages and calculate the dosing rate. A dosing rate ≥80% indicates good compliance. A WeChat group will be established for the participants to improve compliance. The study personnel will post messages in the WeChat group to remind participants to take the samples as scheduled and complete the questionnaire. In addition, each participant will receive a reward of 300 RMB once they complete the follow-up.

Volunteers will be withdrawn from the trial for the following reasons: (1) continuous adverse reactions involving systemic infections that cannot be resolved after certain measures are taken, deleterious metabolic activities, excessive immune stimulation, gene transfer, and gastrointestinal side effects, which may be attributed to probiotics [60]; and (2) patients can actively withdraw from the trial at any time for any reason, and subsequent treatment will not be affected.

### 2.14. Data Collection and Collation

The defecation diary (Figure 2) completed by the volunteers will be collected online daily, and the DASS-21 (Appendix 6) will be collected online as scheduled (see Table 1). One stool sample for the gut microbiome analysis and one for the metabolomics test will be collected from each volunteer on days 0 (preadministration period), 28 (administration period), and 42 (postadministration period); these samples will be stored separately and sent for tests at a temperature of −80°C in a special stool storage kit from Guangdong Longsee Company (https://www.longseemed.com).

An independent data management team (DMT) will be established to maintain and monitor study quality and safety. The data of individual characteristics, defecation diary entries (Figure 2), and DASS-21 data (Appendix 6) completed online by volunteers will be downloaded as Excel spreadsheets for further analysis. The gut microbiome and metabolomic data from stool samples will be examined by the Key Laboratory of Dairy Biotechnology and Engineering, Ministry of Education. All data will be submitted to the DMT for management and monitoring. The DMT will promptly analyse the data and contact collectors to resolve any uncertainty that occurs. All records that contain names or other personal information that could identify a participant, such as ID number or consent forms, will be stored separately from the study records. The database will be password protected by the DMT. The team will also have the authority to conduct an interim analysis or terminate the study's next step if the following situations occur: (1) if the probiotic does not affect the gut microbiome, then the study team will not conduct tests on the faecal metabolomes; and (2) if any severe adverse events related to the probiotics occur, the DMT will suggest terminating the study.

### 2.15. Statistical Analysis

The intention-to-treat (ITT) analysis will be conducted between the two groups. Then, the following four data sets will be used for the per-protocol (PP) analysis: (1) the data from all participants who completed the follow-up and (2) a subgroup analysis regarding levels of compliance (≥80%, 60–80%, and <60%) to validate the robustness of the results. The hypothesis will be tested using the methods described below during the ITT and PP analyses.

First, the Wilcoxon signed-rank sum test will be used for the outcome measures, including the weekly mean frequency of CSBMs, weekly mean stool appearance score, weekly mean difficulty of passing stool score, weekly mean frequency of SBMs, QOL score, and emotional status score, at each observation point to analyse the intergroup differences in each outcome measure. The chi-square (*χ*^2^) test will be used for the outcome measures, namely, the percentage of volunteers with a weekly mean frequency of CSBMs ≥3 and WPUAMA, at each observation point. In the analyses, the *P* value indicating statistical significance will be set to 0.05.

Then, the Kruskal–Wallis rank sum test will be used for multiple comparisons of outcome measures, including the weekly mean frequency of CSBMs, weekly mean stool appearance score, weekly mean difficulty of passing stool score, weekly mean frequency of SBMs, QOL score, and emotional status score, and the *χ*^2^ test will be used for multiple comparisons of outcome measures, including percentage of volunteers with a weekly mean frequency of CSBMs ≥3 and WPUAMA, at each observation point to identify the intragroup changing trends in consecutive phases, namely, pre-, during, and postprobiotic administration. In the analyses, the *P* value indicating statistical significance will be adjusted to 0.05/[*k* (*k* −1)/2], where *k* is the number of sample groups included in the comparison.

In addition, the statistical analyses of metagenomic and metabolomic data will be performed using *R* software (v.4.0.2) and Adobe Illustrator. PCA and PLS-DA will be performed and visualized using the *R* packages vegan, ggplot, and ggpubr, and the adonis *P* value will be generated based on 999 permutations. The *t*-test, Wilcoxon test, and Kruskal–Wallis test will be used to evaluate differences in variables between and within groups; *P* values will be corrected for multiple testing using the Benjamini–Hochberg procedure. Meanwhile, Pearson's and Spearman's correlation coefficients will be calculated to analyse the correlations between different indicators in clinical, metagenomic, and metabolomic data.

## 3. Discussion

Interest in using probiotics as a constipation-relieving agent is increasing, as approximately half of patients have been disappointed with their current treatments. Probiotics hold great promise as a complementary treatment. However, a clear consensus on its recommendation for a patient with constipation is still unavailable because the existing evidence is limited and the methodology used by the existing studies lacks consistency, precluding a comparison of the results. In this context, we developed the protocol for this study.

The key parameters of the methodology used in the previous RCTs and the present RCT are shown and compared in Table 2. The RCTs cited in Table 2 were obtained from recently published systematic reviews or meta-analyses [39–42, 62, 63] that aim to study the effect of probiotics on constipation. Seventy-six RCTs were initially obtained from these systematic reviews or meta-analyses, while 34 RCTs remained after removing 35 duplicate papers and 7 papers that only focused on the effects of probiotics on intestinal flora in healthy adults for the extraction and comparison of key parameters of the methodology (Table 2). As shown in Table 2, the parameters of the methodology are quite inconsistent. For example, the sample size varies substantially from 8 [64] to 453 [65], and the daily dosage ranges from 10^8^ [66] to 10^10^ [67] CFUs; importantly, the diagnostic criteria for constipation, study stage and duration, and outcomes are also obviously different. Under these circumstances, a high-quality RCT protocol is needed to promote the consistency of methodology in future research. Based on the optimized parameters used by the 34 RCTs (Table 2) and our understanding of methodology, we designed the present protocol, of which the parameters are listed in the last row in Table 2.

We optimize the methodology from the following aspects: ① The general principle for a high-quality RCT is strictly obeyed through the participation of third-party independent companies or the DMT, including randomization, parallel control, blinding, allocation concealment, combination of ITT and PP analyses, consideration of withdrawals or dropouts, replication (enough observed study subjects), and other factors. ② The sample size (100 for each group) is obtained based on the expected difference in weekly frequency of CSBMs between the treatment group and control group, which is sufficient to ensure the reliability and accuracy of the results. The sample sizes in 33 of the 35 previous RCTs are dozens or even several participants, which is probably not sufficient to obtain an accurate result. ③ The latest Rome criteria (Rome IV for the present RCT) are suggested for the diagnosis of chronic constipation to avoid any other self-definition by researchers. ④ Multidimensional outcomes have been observed in the present RCT, including improvements in clinical symptoms, QOL score, emotional status score, and modifications in the gut microbiome and faecal metabolome, to produce a chain of evidence from causal improvements in metagenomic and metabolomic data to clinical symptoms and then to the emotional state and QOL. The outcomes are measured using accepted methods, e.g., BSFS [53], PAC-QOL [57], DASS-21 [58], next-generation sequencing, and LC–MS/MS analysis. Thus, the effects of probiotics on chronic constipation in the present study are more reliable and explainable. ⑤ Other key parameters, such as the study stage and duration, are also suggested.

In addition, the protocol is conducted to prove its feasibility and scientificity by studying the effects of *L. plantarum* P9 on chronic constipation. The study is articulated with a clear testable hypothesis stating that *L. plantarum* P9 improves the clinical symptoms and patient QOL by modifying the intestinal flora. Additionally, the safety of *L. plantarum* P9 will be assessed.

Thus, we believe the study will be a high-quality RCT. The results will be more comprehensive than those of existing studies and will objectively and scientifically reflect the effectiveness of *L. plantarum* P9 on constipation. In addition, the protocol will provide methodological guidance for similar studies, avoiding methodological bias and ultimately facilitating the formulation of a consensus on the use of probiotics as a constipation-relieving agent.

### 3.1. Trial Status

This study started recruiting patients on October 1, 2020. To date, 130 patients have been enrolled. Enrolment is expected to be completed by May 31, 2022, and all follow-up visits are expected to be completed by July 31, 2022. The protocol is version 2.0, and the date of the edition is 10 September 2020.

## Figures and Tables

**Table 1:** Study and follow-up schedule.

Item		Screening period (preadministration observation period)		Observation period during administration		Postadministration observation period
		Visit 0	Visit 1	Visit 2	Visit 3	Visit 4
		(Days −14 to −1)	Day 0	Day 14	Day 28	Day 42
Collection of volunteers' basic information	√					
Screening	√					
Signing informed consent form	√					
Stool test (including occult blood)	√					
Defecation diary	Completed online daily					
Primary outcomes	Weekly mean frequency of CSBMs		√	√	√	√

Secondary outcomes	Weekly mean frequency of CSBMs ≥3		√	√	√	√
	Weekly mean frequency of SBMs		√	√	√	√
	Weekly mean stool appearance score		√	√	√	√
	Weekly mean difficulty of passing stool score		√	√	√	√
	Weekly mean stool appearance score		√	√	√	√
	Weekly mean difficulty of passing stool score		√	√	√	√
	WPUAMA		√	√	√	√
	Quality-of-life score		√	√	√	√
	Emotional state score		√	√	√	√
	Gut microbiome		√		√	√
	Faecal metabolome		√		√	√

Safety measures	Adverse events (AEs)			√	√	√
	Serious adverse events (SAEs)			√	√	√

Verification of compliance with the intervention					√	
Concomitant medications			√	√	√	√

*Note*. Except for the quality-of-life score and emotional state score, the other outcome measures are calculated from the defecation diary.

**Table 2:** The comparisons of methodologies among the present and previous RCTs related to the effect of probiotics on chronic constipation.

Study	Design	Allocation concealment/Blinding/ITT analysis/Description of withdrawals or dropouts	Sample size (probiotics:control)	Population	Age (years)	Diagnostic criteria	Intervention	Comparator	Probiotic strain	Outcomes	Daily dosage (CFUs)	Study stage (duration)
Agrawal [68]	Randomized, double-blind, controlled, parallel group study	Yes/Yes/Yes/Yes	17/17	Irritable bowel syndrome with constipation in females	20–69	Rome III criteria for constipation predominant IBS	A fermented milk containing probiotics	A milk-based nonfermented dairy product	*Bifidobacterium lactis*	Abdominal distension, gastrointestinal transit, abdominal symptoms, and bowel habit	1.25 × 10^10^	Screening period/baseline (11 days), administration period (28 days), postadministration period (7 days)
Holma [64]	Randomized, controlled, unblinded, 2 × 2 factorial design	Unclear/Unclear/No/No	10/12/11/10/8^a^	Adults with self-reported constipation	22–78	<5 defecations/week without laxatives or <7 defecations/week with laxatives, and self-reported constipation	Whole-grain rye bread or *Lactobacillus rhamnosus* GG (LGG) or whole-grain rye bread + LGG	Laxatives or white wheat bread	Cultured buttermilk supplemented with *Lactobacillus* GG	Faecal weight, pH, short-chain fatty acids (SCFA) and bacterial enzyme activities, total intestinal transit time (TITT), and breath hydrogen	2 × 10^10^	Baseline period (1 wk)-intervention period (3 wks)
Hongisto [69]	Randomized, controlled, 2 × 2 factorial design	No/No/No/No	14/15/16/14^b^	Women with self-reported constipation	18–57	Feelings of reduced/less-frequent bowel movements, as well as straining at defecation	Fibre-rich rye bread + *Lactobacillus rhamnosus* GG (LGG)	Fibre-rich rye bread, LGG, or low-fibre toast	*Lactobacillus* GG	TITT, faecal frequency and consistency, difficulty in defecation, and gastrointestinal symptoms	1.5 × 10^10^	Screening/baseline period (1 wk)-administration period (3 wks)-postadministration period (3 wks)
Malpeli [70]	Randomized, double-blind, placebo-controlled and crossover	Yes/Yes/No/Yes	28/35	Healthy women	21–60	Those with a slow transit perception and/or abdominal pain (bloating) or slow transit (functional constipation) according to Rome III criteria	The synbiotic yoghurt contained the test probiotics	The standard yoghurt	*Bifidobacterium infantis* and *Lactobacillus casei*	Intestinal transit time, voiding frequency, stool consistency and bloating, intestinal flora	10^9^-10^10^	Screening/baseline period (15 days)-administration period (15 days)-washout period (15 days)-administration period (15 days)
Waller [71]	Sex-stratified, triple-blind, placebo-controlled, parallel-group, dose-ranging study	Unclear/Yes/No/Yes	33/33/34^c^	Adults with constipation	25–65	Self-report of stool type 2–4 on the Bristol Stool Chart and an average of 1–3 bowel movements per week.	The capsules contained the test probiotics	Placebo	*Bifidobacterium lactis*	Food frequency, whole gut transit time, functional gastrointestinal symptom frequency	17.2 × 10^9^ or 1.8 × 10^9^	Screening/baseline period (7 days)-administration period (14 days)
Krammer [72]	Randomized double-blind placebo-controlled trial	Unclear/Yes/No/No	12/12	Female adults with chronic constipation	Unclear (∼50)	Transit time >72 h.	Fermented milk drink containing test probiotics	Placebo	*Lactobacillus casei* Shirota (LcS)	Colonic transit time, stool frequency and consistency, constipation-related and gastrointestinal symptoms	6.5 × 10^9^	Screening/baseline period (2 wks)-administration period (4 wks)- postadministration period (4 wks)
Bazzocchi [73]	Randomized double-blind, controlled trial	Yes/Yes/No/No	17/12	Patients with severe functional constipation	19–65	Constipation consecutively matching the Rome III diagnostic criteria for functional constipation	A synergic mixture of the prebiotic psyllium fibre and five probiotic strains	Maltodextrin	*L. plantarum*, *L. acidophilus* and *L. rhamnosus* and *B. longum spp. longum* and *B. breve* species	% Bowel motions with normal stools, decrease in Agachan–Wexner score for constipation severity, increase in faecal levels of *Lactobacillus* and *Bifidobacterium*	unclear	Screening/baseline period (2 wks)-administration period (8 wks)
Cudmore [74]	Randomized, double-blind, placebo-controlled clinical study	Yes/Yes/Yes/Yes	35/34	Adults with chronic, functional constipation	18–80	Rome III diagnostic criteria for functional constipation	5 g sachet containing test probiotics	Placebo	*L. rhamnosus*, *B. bifidum*, *L. acidophilus*, *L. plantarum*, *Lactobacillus bulgaricus*	Number of bowel movements, stool consistency, quality of life, constipation symptoms, reduced laxative use by the subjects	1.2 × 10^9^	Screening/baseline period (14 d)-administration period (7 d)
Eskesen [65]	Randomized, double-blind, placebo-controlled	Yes/Yes/Yes/No	343/452/453^d^	Healthy subjects with constipation	18–70	Low defecation frequency (2–4 times/week) and complaints of general abdominal discomfort	Probiotic strain in capsule form	Placebo	*Bifidobacterium animalis* subsp. *Lactis*	Defecation frequency and gastrointestinal well-being responder rates, symptom severity scores for abdominal pain and bloating	1 × 10^9^ or 10 × 10^9^	Screening/baseline period (2 wks)-administration period (4 wks)
Mazlyn, [75]	Randomized, double-blind, placebo-controlled	Unclear/Yes/yes/Yes	47/43	Adults with functional constipation	18–60	Rome II criteria	Shirota fermented milk containing the test probiotics	Placebo	*Lactobacillus casei* strain Shirota	Constipation severity, stool frequency, stool consistency and quantity	3.0 × 10^10^	Screening/baseline period (2 wks)-administration period (4 wks)
Ojetti [76]	Randomized, double-blind, placebo-controlled	Unclear/Yes/yes/Yes	20/20	Adults with functional constipation	Unclear (approximately 36 ± 15)	Rome III	Probiotic tablets containing the test probiotics	Placebo	*Lactobacillus reuteri*	Bowel movements/week frequency, stool consistency according to BSS	2 × 10^8^	Administration period (4 wks)
Tanaka [77]	Randomized, double-blind, placebo-controlled	Unclear/Yes/Unclear/Yes	18/20	Adults with constipation	25–59	Frequency of bowel movements of <5.0 times/week assessed using a questionnaire	Milk-like drink containing test probiotics	Placebo	*B. animalis* subsp. *lactis*	Intestinal *Bifidobacteria*, frequency of defecation	1.5 × 10^10^	Screening/baseline period (2 wks)-administration period (8 wks)
Waitzberg [78]	Randomized, double-blind, placebo-controlled	Unclear/Yes/Unclear/Yes	50/50	Constipated adult women	18–75	Rome III	Synbiotic containing multiple probiotics	Placebo	*L. paracasei*, *L. rhamnosus*, *L. acidophilus*, *B. lactis*	Stool frequency, consistency and shape, abdominal pain, bloating and flatulence, constipation intensity	10^8^-10^9^	Screening/baseline period (1 wk)-administration period (30 days)
Yang [79]	Randomized, placebo-controlled	Unclear/Unclear/Yes/No	59/56	Adult females with constipation	25–65	Less than three stools per week, increased stool hardness, nonorganic constipation and habitual constipation	Fermented milk contains probiotics	Acidified milk	*B. lactis*	Stool frequency, defecation condition scores, stool consistency and food intake, safety evaluation	1.25 × 10^10^	Screening/baseline period (1 wk)-administration period (2 wks)
Koebnick [80]	Randomized, double-blind, placebo-controlled	Unclear/Yes/Unclear/No	35/35	Adults with chronic idiopathic constipation	18–70	NA	Probiotic beverage	Placebo	*L. casei* strain Shirota	Severity of constipation, defecation frequency, stool consistency, occurrence and degree of flatulence, occurrence, and degree of bloating	6.5 × 10^9^	Screening/baseline period (2 wks)-administration period (4 wks)
Bu [81]	Randomized, double-blind, placebo-controlled	Yes/Yes/Yes/Yes	18/18/9^e^	Children with chronic constipation	<10	Stool frequency of <3 times per week for >2 months and at least one of the following minor criteria: anal fissures with bleeding due to constipation, faecal soiling, or passage of large and hard stool	Capsules containing probiotics	Magnesium oxide (traditional laxative) or placebo	*L. casei rhamnosus*	Frequency of daily bowel movements, stool consistency, abdominal pain, faecal soiling, intestinal flora	8 × 10^8^	Administration period (4 wks)
Banaszkiewicz [82]	Randomized, double-blind, placebo-controlled	Yes/Yes/Yes/No	43/41	Children with constipation	2–16	<3 spontaneous bowel movements per week for at least 12 weeks	Lactulose with *Lactobacillus* GG	Lactulose with placebo	*L. rhamnosus* GG	Greater than or equal to 3 spontaneous BMs per week with no episodes of faecal soiling, the number of BMs per week, number of episodes of faecal soiling per week, stool consistency, and straining frequency per week, percentage of patients using laxatives was assessed at 24 weeks	2 × 10^9^	Administration period (12 wks)-postadministration period
Bouvier [83]	Double blind, placebo-controlled parallel study	Yes/Yes/No/No	36/36	Healthy adults	21–42	Normally indicated by medical examination and not taking any medication for at least four weeks	Fermented milk containing the test probiotics	Placebo milk	*B. animalis*	Colonic transit time	9.75 × 10^10^	Screening/baseline period (10 days)-administration period (11 days)
Marteau [84]	Double-blind, randomized, controlled study	Unclear/Yes/No/No	17/15	Healthy women	18–45	Judged by a medical examination	Fermented milk containing the probiotics	Fermented milk	*B. animalis* strain	Total and sigmoid transit times, the other transit times, faecal weight, pH, bacterial mass, and bile acids	0.19–1.9 × 10^10^	Screening/baseline period (10 days)-administration period (10 days)-washout period (10 days)-administration period (10 day)
Merenstein [85]	Triple-blind, placebo-controlled, two-period crossover trial	Yes/Yes/Yes/Yes	34/34	Women	18–65	Self-reported history of straining during bowel movements or hard or lumpy stools in the past 2 years	Yoghurt containing the test probiotics	Yoghurt	*B. animalis ssp. lactis*	Colonic transit time, the number of bowel movements/week, QOL, frequency of bowel movements over 2 weeks, frequency of constipated stools, % positive for *B. animalis* ssp*. Lactis*, daily diet, compliance	2.0–5.6 × 10^10^	Screening/baseline period (duration is unclear)-administration period (14 days)-washout period (6 wks)-administration period (14 days)
Ishizuka [86]	Placebo-controlled double-blind, crossover	Unclear/Yes/Yes/Yes/No	12/12	Adults suffering from constipation	20–23	Number of defecations is less than or equal to 5.0 times/week	Milk-like drink containing the test probiotics	Milk-like drink	*B. lactis*	Intestinal *Bifidobacteria*	1 × 10^10^	Screening/baseline period (2 wks)-administration period (2 wks)-washout period (2 wks)-administration period (2 wks)
Del Piano [87]	Randomized, double-blind, placebo-controlled study	Unclear/Yes/Yes/Yes/Yes	80/110/110^f^	Healthy volunteers with evacuation disorders and hard stools	24–71	Judged by a complete physical examination, normal values of laboratory tests, and no evidence of gastrointestinal disease on plain abdominal X-ray and ultrasound	A half glass of water containing the test probiotics	Placebo	Mixture of *L. plantarum* and *B. breve* or *B. animalis* subspecies *lactis*	Ease of expulsion, number of weekly evacuations, anal itching, burning, and pain abdominal bloating, sensation of complete emptying	5 × 10^9^ or 2.5 × 10^9^	Screening/baseline period (7 days)-observation period during administration (30 days)
Riezzo [88]	Randomized, double-blind, placebo-controlled	Unclear/Yes/No/Yes	28/28	Adults with functional constipation	19–65	Rome III criteria for FC without matching Rome criteria for IBS	*L. reuteri* DSM 17938	Placebo	*L. reuteri*	CSS, PAC-QOL	2 × 10^8^ and 4 × 10^8^	Screening/baseline period (7 days)-observation period 1 during administration (15 days with the high dosage)-observation period 2 during administration (90 days with the low dosage)
Riezzo [89]	Randomized, double-blind, crossover study	Unclear/Yes/Yes/Yes	10/10	Adults with functional constipation	19–70	Rome Criteria III for constipation 20, Constipation Scoring System (CSS)	Probiotic-enriched artichokes	Artichokes	*L. paracasei*	Stool consistency, GSRS sum score, SCFAs, colonic transit time	2 × 10^10^	Screening/baseline period (7 days)-observation period during administration (15 days)-washout period (4 wk)-observation period during administration (15 days)
Dimidi [46]	Randomized, double-blind, placebo-controlled	Unclear/Yes/Yes/Yes	37/38	General population with mild constipation	18–65	Modified Rome III diagnostic criteria for functional constipation	Milk powder with probiotics	Placebo milk powder	*B. lactis*	Whole gut transit time, regional gut transit time, constipation severity, stool frequency and stool consistency, QOL, gut microbiota composition, safety outcomes	1.5 × 10^10^	Screening/baseline period (duration is unclear)-observation period during administration (4 wks)-postadministration observation period (8 wks)
Mirghafourvand [90]	Randomized, triple-blind, placebo-controlled	Unclear/Yes/No/Yes	29/28	Constipated pregnant women with a gestational age of 24–28 weeks	>18	Rome III criteria	Probiotic yoghurt containing the test probiotics	Conventional yoghurts	*L. acidophilus* and *B. lactis*	The defecation frequency, stool consistency, straining during defecation, sensation of anorectal obstruction, sensation of incomplete evacuation and manual manoeuvres to facilitate defecation, the amount of defecation, stool colour, and QOL	4.8 × 10^8^	Screening/baseline period (duration is unclear)-observation period during administration (4 wks)-postadministration observation period (2 wks)
Favretto [66]	Randomized controlled trial	Unclear/No/No/No	15/15	Constipated women	20–60	Rome III consensus	Fresh cheese containing the test probiotics	Regular fresh cheese	*B. lactis*	Symptoms of Rome III criteria	1 × 10^8^	Screening/baseline period (duration is unclear)-observation period during administration (30 d)
De Paula [91]	Open, randomized, controlled study in parallel groups with intercrossing	Yes/No/No/Yes	Varied according to different outcomes	Women with functional constipation (266) and women without constipation (112)	18–55	Rome II criteria	Dessert with probiotics	Lacteous dessert	*B. animalis*	Stool frequency >5/week, stool frequency >3/week, stool shape, straining effort and pain during bowel evacuation, stool frequency (bowel movements/week), stool shape, pain and straining effort associated with bowel evacuation	1 × 10^8^	Screening/baseline period (duration is unclear)-observation period during administration (2 wks)-observation period during cross-administration (2 wks)
Ding [92]	Prospective Randomized Trial	Yes/Yes/No/Yes	48/45	Patients with slow transit constipation	<18	Rome III criteria for chronic constipation	Synbiotic containing the test probiotics	Placebo	Unclear	Stool frequency and consistency, colonic transit time (CTT), evacuation and abdominal symptoms, patient assessment of constipation symptoms, gastrointestinal QOL index scores, satisfaction scores, and adverse events	unclear	Screening/baseline period (duration is unclear)-observation period during administration (12 wks)
Fateh [93]	Double-blind, randomized, placebo-controlled trial	Unclear/Yes/No/no	31/29	Young men suffering from functional constipation	>18	Rome III criteria for chronic constipation	Synbiotic mixture of probiotics	Capsules	*L. casei*, *L. rhamnosus*, *Streptococcus thermophilus*, *B. breve*, *L. acidophilus*, *B. longum*, *L. bulgaricus*	Stool frequency per week, overall score of patient assessment of constipation symptoms, the Bristol Stool Form Scale, abdominal symptoms score, rectal symptoms score, stool symptoms score	1 × 10^8^	Screening/baseline period (duration is unclear)-observation period during administration (4 wks)
Jayasimhan [94]	Randomized, double-blind, placebo-controlled	Yes/Yes/Yes/Yes	50/58	Constipated adults	18–81	Rome III criteria for chronic constipation	Microbial cell preparation containing probiotics	Placebo	*L. acidophilus*, *L. casei*, *L. lactis*, *B. bifidum*, *B. longum* and *B. infantis*	Frequency of bowel movements per week, self-perception of the improvement in symptoms (straining, lumpy or hard stool, sensation of incomplete evacuation, sensation of anorectal blockage and manual manoeuvres to aid in defecation)	3 × 10^10^	Screening/baseline period (duration is unclear)-observation period during administration (1 wk)
Magro [95]	Randomized, double-blind, controlled study	No/Yes/No/Yes	21/26	Individuals with chronic constipation	18–45	Rome III criteria for chronic constipation	Yoghurt containing probiotics	Yoghurts	*L. acidophilus* and *B. lactis*	Agachan's score, bowel movements/day, colonic transit time (hours)	>1 × 10^9^	Screening/baseline period (duration is unclear)-observation period during administration (2 wks)
Kondo, [67]	Double-blind, placebo-controlled, parallel-group design	Yes/Yes/No/Yes	32/34	Elderly patients with constipation	>65	Unclear	Powder containing probiotics	Placebo powder	*B. longum*	Times of defecation, stool form, and consistency	2.5 × 10^10^	Screening/baseline period (1 wk)-observation period during administration (16 wks)
			32/37/33^g^	Elderly patients with constipation	>65	Unclear	Powder containing probiotics	Powder	*B. longum*	Bowel movements, faecal microbiota, stool form, and consistency	2.5 × 10^10^ or 5 × 10^10^	Screening/baseline period (1 wk)-observation period during administration (16 wks)
Yoon [96]	Randomized, double-blind, placebo-controlled Study	No/Yes/No/Yes	90/90	Adults with chronic constipation	18–75	Rome IV criteria	Chocolate case containing probiotics	Placebo	*S. thermophilus* and *L. plantarum*	Faecal microbiota, global improvement scale, frequency of bowel movement, Bristol Stool Form Scale and Complete Spontaneous Bowel Movements (CSBM), Gastrointestinal Symptom Rating Scale, health-related QOL	3.0 × 10^8^ and 1.0 × 10^8^	Screening/baseline period (1 wk)-observation period during administration (4 wks)-postadministration observation period (4 wks)
The present study	Randomized, double-blind, placebo-controlled clinical trial	Yes/Yes/Yes/Yes	100/100	Adults with chronic constipation	18–65	Rome IV criteria	Powder containing probiotics	Placebo	*L. plantarum* P9	Frequency of CSBMs per week, weekly mean frequency of CSBMs >3, weekly mean frequency of SBMs, weekly mean stool appearance score, weekly mean difficulty of passing stool score, WPUAMA, QOL score, emotional status score, gut microbiome, and faecal metabolome	>1 × 10^11^	Screening/baseline period (2 wks)-observation period during administration (4 wks)-postadministration observation period (2 wks)

*Notes*: The sample sizes correspond to the following groups: ^a^groups receiving rye bread, LGG, rye bread + LGG, control, and laxative, respectively; ^b^the groups receiving rye bread + LGG, rye bread, LGG, and control, respectively; ^c^groups receiving the high dosage, low dosage, and placebo, respectively; ^d^groups receiving the low dosage, high dosage, and placebo, respectively; ^e^groups receiving MgO, probiotics, placebo, respectively; ^f^groups receiving placebo, probiotics1, and probiotics2, respectively; and ^g^groups receiving the low dosage, high dosage, and control, respectively.

## Data Availability

Data sharing is not applicable to this trial as no database is generated or analysed for the current study. And when the study is completed, the study results will be released to the public, volunteers, and the general medical community via publishing a journal article, with all related data being available.

## Supplementary Materials

Supplementary Materials Appendix 1: trial registration data set. Appendix 2: copy of the Ethical Approval Document. Appendix 3: Bristol Stool Form Scale. Appendix 4: informed consent materials. Appendix 5: Patient Assessment of Constipation Quality-of-Life (PAC-QOL) questionnaire. Appendix 6: Depression, Anxiety and Stress Questionnaire. Appendix 7: National Natural Science Foundation of China.
