# Supplementary material for: Effects of Lactobacillus plantarum P9 Probiotics on Defecation and Quality of Life of Individuals with Chronic Constipation: Protocol for a Randomized, Double-Blind, Placebo-Controlled Clinical Trial
Source: Evid Based Complement Alternat Med. 2022 Jun 13;2022:4144321. doi: 10.1155/2022/4144321 (PMC9208957; doi:10.1155/2022/4144321)
Supplement: Supplementary Materials — Appendix 1: trial registration data set. Appendix 2: copy of the Ethical Approval Document. Appendix 3: Bristol Stool Form Scale. Appendix 4: informed consent materials. Appendix 5: Patient Assessment of Constipation Quality-of-Life (PAC-QOL) questionnaire. Appendix 6: Depression, Anxiety and Stress Questionnaire. Appendix 7: National Natural Science Foundation of China. [file 4144321.f1.docx]

**Appendixes**

**Appendix 1 Trial registration dataset**

| **Data category** | **Information** |
| --- | --- |
| Primary registry and trial identifying number | http://www.chictr.org.cn/showproj.aspx?proj=54024  ChiCTR2000038396 |
| Date of registration in primary registry | 22 September, 2020 |
| Source(s) of monetary or material support | The trial was supported by funding from the National Natural Science Foundation of China (31720103911) and Jiangzhong Pharmaceutical Co., Ltd. to Heping Zhang. |
| Primary sponsor | Jiangzhong Pharmaceutical Co., Ltd. |
| Public title | Effect of *Lactobacillus plantarum* P9 on defecation, quality of life, and gut microbiota in patients with chronic constipation |
| Scientific title | *Effect of Lactobacillus plantarum P9 on defecation, quality of life, and gut microbiota in patients with chronic constipation* |
| Countries of recruitment | China |
| Health condition(s) or problem(s) studied | Chronic constipation; Probiotics |
| Intervention(s) | ***Probiotics group:*** *Volunteers will take L. plantarum P9 powder directly or with warm water (below 40℃) on a full stomach, 1 pack (100 billion CFUs) per day.*  ***Placebo group:*** *Volunteers will take the placebo in the same manner as the probiotics group. The placebo contains no probiotics and comprises maltodextrin (60%), orange powder (20%), and maltitol (20%) with the same appearance, packaging, and taste as the L. plantarum P9 powder.* |
| Key inclusion and exclusion criteria | **Inclusion criteria:** 1. The following symptoms occurred at least 6 months before enrolment, and two or more of the following symptoms were experienced in the previous 3 months:  a. Bowel movements were laboured at least 25% of the time.  b. Massive or hard stools occurred at least 25% of the time (refer to type 1-2 assessed using the Bristol faeces scale).  c. Defecated at least 25% of the time.  d. A sense of anal obstruction or obstruction in at least 25% of defecations.  e. At least 25% of the time, defecation required manual assistance (e.g., finger assistance or pelvic floor support).  f. Spontaneous defecation occurred less than 3 times per week.  (2) Loose stools were rare without laxatives.  2. Participants are also required to sign an informed consent form and meet one of the following two criteria:  (1) Age 18 to 50 years, males and females, with a routine stool routine examination (including occult blood) report during the screening period that was normal or abnormal results were deemed to be of no clinical significance by the investigator.  (2) Age 18 to 50 years with normal colonoscopy results reported by a domestic tertiary hospital within the past 6 months or abnormal results with no clinical significance determined by researchers. |
|  | **Exclusion criteria:** 1. A family history of colon cancer, coeliac disease, or inflammatory bowel disease.  2. Colonoscopy-confirmed organic intestinal diseases.  3. Recent preparations for pregnancy (including males and females), pregnant or lactating women.  4. Allergies to samples or ingredients.  5. Use of antibiotics or probiotics in the past two weeks.  6. Use of antianxiety, antidepression or other psychiatric drugs in the past month.  7. Need for long-term drugs to improve constipation.  8. Diagnosed with myocardial infarction, cerebral infarction, malignant tumour or other serious diseases that researchers believe are not suitable for inclusion.  9. Subjects with major mental disorders, difficulty in controlling their actions and inability to cooperate.  10. Illiterate, unable to understand the informed consent form, and unable to sign the informed consent form by themselves. |
| Study type | Interventional |
|  | Allocation: randomized intervention model. Parallel assignment masking: double blind (subject, investigator, caregiver, and outcomes assessor) |
|  | Primary purpose: prevention |
| Date of first enrolment | October 1, 2020 |
| Target sample size | 200 |
| Recruitment status | Recruiting |
| Primary outcome(s) | The primary outcome is the average number of complete spontaneous bowel movements (CSBMs) weekly. |
| Key secondary outcomes | The secondary outcome measures include the proportion of average CSBMs per week ≥3 times; the number of weekly average spontaneous bowel movements (SBMs); average weekly stool morphology score according to the Bristol scale; average weekly score of difficulty in defecating; number and proportion of weekly assisted bowel movements; Quality of Life score (using Patient-Assessment of Constipation Quality of Life, PAC-QQL); emotional state score, gut microbiome and faecal metabolome. |

**Appendix 2 Copy of the Ethical Approval Document with English translation.**


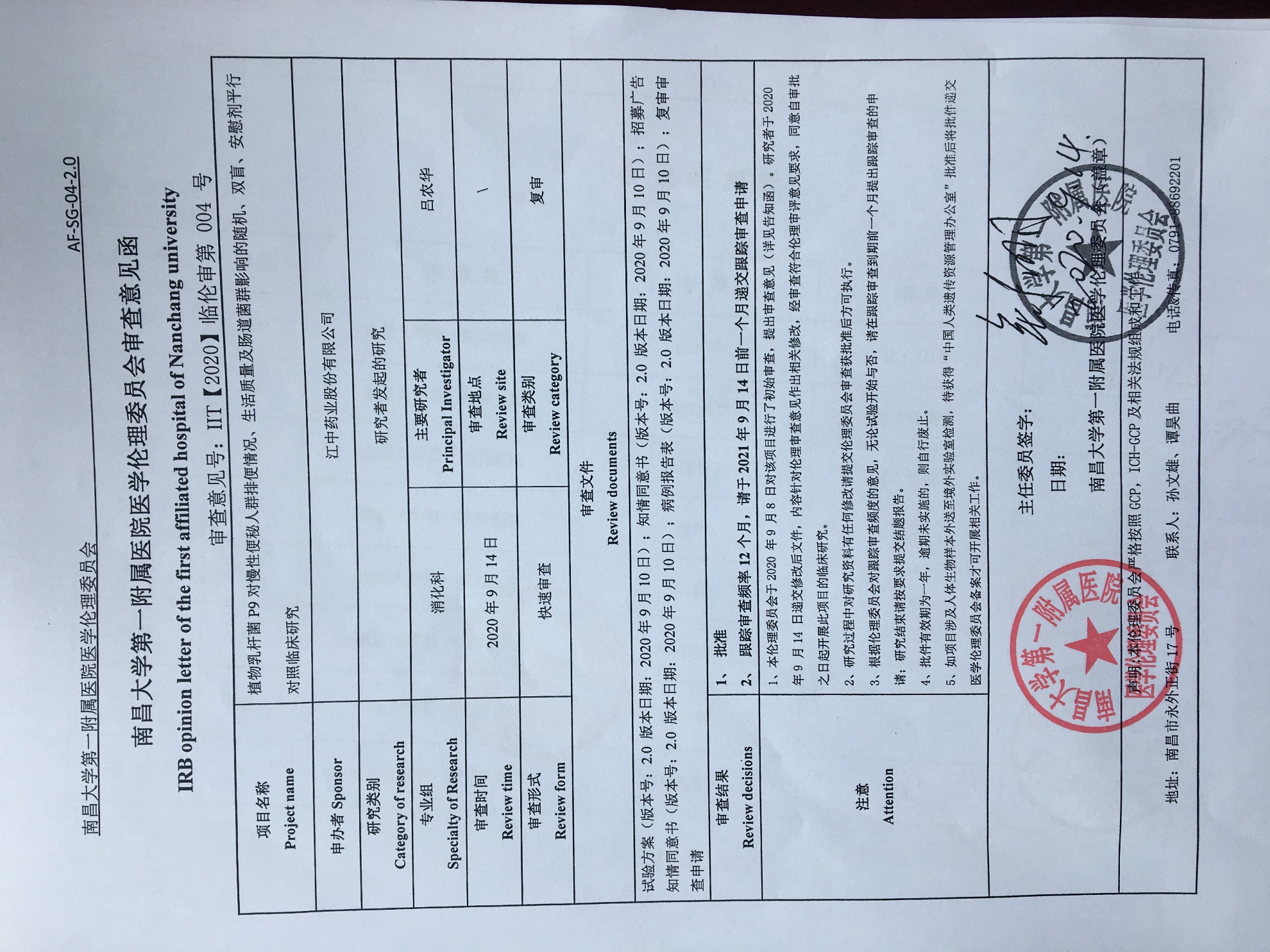


Medical Ethics Committee of the First Affiliated Hospital of Nanchang University AF-SG-04-2.0

**IRB opinion letter of the First Affiliated Hospital of Nanchang University**

Review opinion number: IIT [2020] Clinical Ethics Review No. 004

| Project name | Effect of *Lactobacillus plantarum* P9 on defecation, quality of life and gut microbiome in individuals with chronic constipation: A randomized, double-blind, placebo-controlled clinical trial | | |
| --- | --- | --- | --- |
| Sponsor | Jiangzhong Pharmaceutical Co., Ltd. | | |
| Category of research | investigator-initiated clinical trial | | |
| Specialty of Research | Gastroenterology | Principal Investigator | Nonghua Lu |
| Review time | September 14, 2020 | Review site | \ |
| Review form | Quick review | Review category | Reexamination |
| Review documents | | | |
| Clinical trial protocol（Version no.: 2.0 Version Date: 10 September 2020); Informed consent (Version no.: 2.0 Version Date: 10 September 2020); Recruitment advertisement (Version no.: 2.0 Version Date: 10 September 2020); Case Report Form (Version no.: 2.0 Version Date: 10 September 2020); Application for review | | | |
| Review decisions | 1. Approve 2. The frequency of follow-up review is 12 months. Please submit the application for follow-up review one month before September 14, 2021 | | |
| Attention | 1. The ethics committee conducted an initial review of the project on September 8, 2020 and proposed some corrections (see the notification letter for details). The researcher submitted the revised documents on September 14, 2020, and the content was revised in response to the ethical review board opinions. Reexamination found that the revised documents met the requirements of the ethical review board opinion; thus, the clinical research of this project was approved to start from the date of approval. 2. Any change to the research program during the study should be submitted to the ethics committee for review and requires approval before implementation. 3. According to the suggestion of the ethics committee on the frequency of follow-up review, regardless of whether the trial has started, please submit an application for follow-up review one month before the expiration of the follow-up review. After completing the research, please submit a final report as needed. 4. The approval document is valid for one year. If the trial is not implemented within the time limit, approval will be automatically annulled. 5. If the project involves sending biological samples from human bodies to overseas laboratories for testing, an application must be submitted to the Medical Ethics Committee for record, and the approval of the “China Human Genetic Resources Management Office” must be obtained before the relevant work is conducted. | | |
| Chairman's signature: Ming Shu  Date: September 14, 2020  Medical Ethics Committee of the First Affiliated Hospital of Nanchang University ([seal](file:///C:/Users/13424/AppData/Local/youdao/dict/Application/8.9.6.0/resultui/html/index.html#/javascript:;)): | | | |
| Statement: The ethics committee is founded and conducts work in strict accordance with GCP ICH-GCP and related regulations.  Address: No. 17 Yongwaizheng Street, Nanchang City Contact: Sun Wenxiong, Tan Haoqu Tel & Fax: 0791-88692201 | | | |


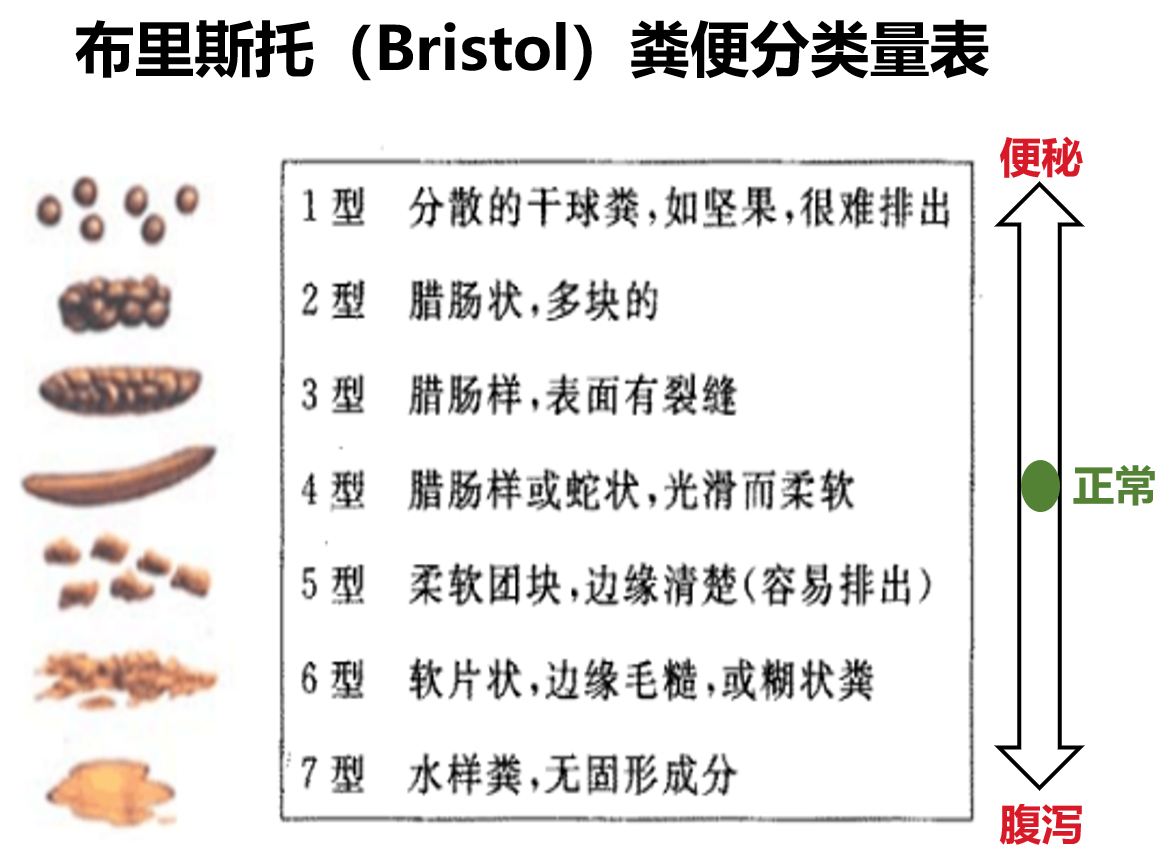


**Type 5: Somewhat loose: soft blobs with clear edges (easy to pass)**

**Type 1: Separate hard lumps, like nuts (difficult to pass)**

**Diarrhoea**

**Type 7: Watery: no solid pieces**

**Type 6: Runny: fluffy pieces with ragged edges, or a mushy stool**

**Type 4: Normal consistency: sausage- or snake-like; smooth and soft**

**Type 3: Sausage-like, with cracks on the surface**

**Type 2: Sausage-shaped, lumpy**

**Constipation**

**Normal**

**Appendix 3** **Bristol Stool Form Scale**

**Appendix 4 Informed consent materials.**

**Effect of *Lactobacillus plantarum* P9 probiotics on defecation and quality of life of individuals with chronic constipation: Protocol for a randomized, double-blind, placebo-controlled clinical trial**

**Patient informed consent**

Dear patient, you will be invited to participate in a clinical study. This study is jointly sponsored by the First Affiliated Hospital of Nanchang University, Inner Mongolia Agricultural University, and Jianzhong Pharmaceutical Co., Ltd. The study is a randomized, double-blind, placebo, parallel-controlled clinical study of the effects of *Lactobacillus plantarum* P9 on defecation and quality of life in people with chronic constipation.

We hereby explain the following content:

**1. Background and objective**

Constipation is a common diagnosis made by gastroenterologists based on the assessment of infrequent bowel movements (<3 per week) and difficult stool passage. Patients may have multiple complaints, including a sense of incomplete defecation, abdominal pain, bloating, and excessive straining accompanied by sensation of anorectal blockage during stool passage, requiring manual assistance. The global prevalence of constipation ranges from 10% to 30%. Patients may constantly suffer from physical symptoms and psychological distress, and chronic constipation might disturb people’s lives, study, and work due to dyspareunia, sexual dysfunction, urine retention, reduced mental health and social function, school absenteeism, loss of work days and the cost of medical care.

Probiotics may be a treatment to address these problems. However, clinical trials pinpointing the effectiveness of probiotics for chronic constipation are currently limited. In this specific context, we designed and conducted this study.

**2. Introduction of *Lactobacillus plantarum* P9**

“Lihuo Probiotic Solid Beverages” is a probiotic product that has been marketed by Jiangzhong Pharmaceutical Co., Ltd. and is composed of *Bifidobacterium lactis* V9, *Lactobacillus casei* Zhang, and *L. plantarum* P9. *L. plantarum* P9 is one of the three strains of probiotic in beverages already on the market. *L. plantarum* P9 is a probiotic strain isolated from a naturally fermented sour porridge in Bayannaoer, Inner Mongolia. Previous studies have shown that *L. plantarum* P9 regulates the intestinal flora, increases the abundance of beneficial bacteria, and decreases the abundance of harmful bacteria.

**3. Criteria for participating the trials**

Trained study implementers and clinical specialists will discuss the criteria for participating in the trials with you and the information provided in the video and information sheets. Please consider the following information to determine whether you are eligible to participate in the trial:

Inclusion criteria

Eligible patients should fully meet the following criteria:

(1) Onset of the following symptoms for at least 6 months before enrolment and symptoms within the past 3 months:

1) Two or more of the following symptoms: a. Difficulty passing stool in at least 25% of defecations; b. Lumpy or hard stool in at least 25% of defecations (Bristol types 1 or 2 (Appendix 1)); c. Incomplete defecation in at least 25% of the defecations; d. Sense of anorectal obstruction in at least 25% of defecations; e. Need for manual assistance for defecation (such as using fingers to assist with defecation or pelvic floor support) in at least 25% of defecations; and f. Fewer than 3 spontaneous bowel movements (SBMs) per week.

2) Loose stool rarely occurs without the use of laxatives.

3) Insufficient stools are rarely present without the use of laxatives.

(2) Willing to sign the informed consent form.

(3) The volunteers involved in this study will be patients with chronic constipation aged 18-65 years. For patients aged from 18 (exclusive) to 50 (inclusive) years, the result of stool tests (including occult blood) conducted during the screening period must be normal or abnormal but judged by the investigators as clinically irrelevant. For patients aged from 50 (exclusive) to 65 (inclusive) years, the result of colonoscopy performed at a tertiary or higher-level hospital within the past 6 months must be normal or abnormal but determined clinically irrelevant by the investigators. All participants willing to participate in this trial must sign the informed consent form.

Exclusion criteria

Volunteers with any of the following conditions will be excluded:

(1) Personal or family history of colon cancer, celiac disease, or inflammatory bowel disease.

(2) Intestinal organic diseases confirmed with previous colonoscopy.

(3) Plans to become pregnant or father a child in the next 3 months, or pregnancy or breastfeeding in women.

(4) Allergies to samples or ingredients.

(5) Use of antibiotics or probiotics within the past two weeks.

(6) Use of antianxiety, antidepressant, or other psychotropic drugs within the past month.

(7) Need for long-term use of medications for constipation.

(8) History of severe diseases, such as myocardial infarction, cerebral infarction, and malignant tumour, judged by the investigators as disqualifying conditions.

(9) Major mental illnesses, inability to control one’s actions, or inability to cooperate.

(10) Illiteracy, inability to understand the informed consent form, or inability to independently sign the informed consent form.

**4. Research groups and interventions**

If you agree to participate in this study, please sign this informed consent form. The study will include three phases, including screening (a preadministration observation period), an administration observation period, and a postadministration observation period. The interventions in each phase are described below.

(1) Screening period (preadministration observation period of preadministration) (days -14 to 0): After signing an informed consent form, you will undergo a 14-day screening period when you will not be allowed to take any medicines or health products to improve constipation symptoms. Additionally, you will be asked to collect one stool sample and complete an online diary daily (Figure 1). At the end of the screening period, the diary and stool exam results will be reviewed and used to determine whether you are eligible according to the inclusion and exclusion criteria. The steps described below will be applied for the formal intervention and follow-up visits.

A random sequence will be generated by the computer and used to randomly assign you to a probiotic group or a placebo group.


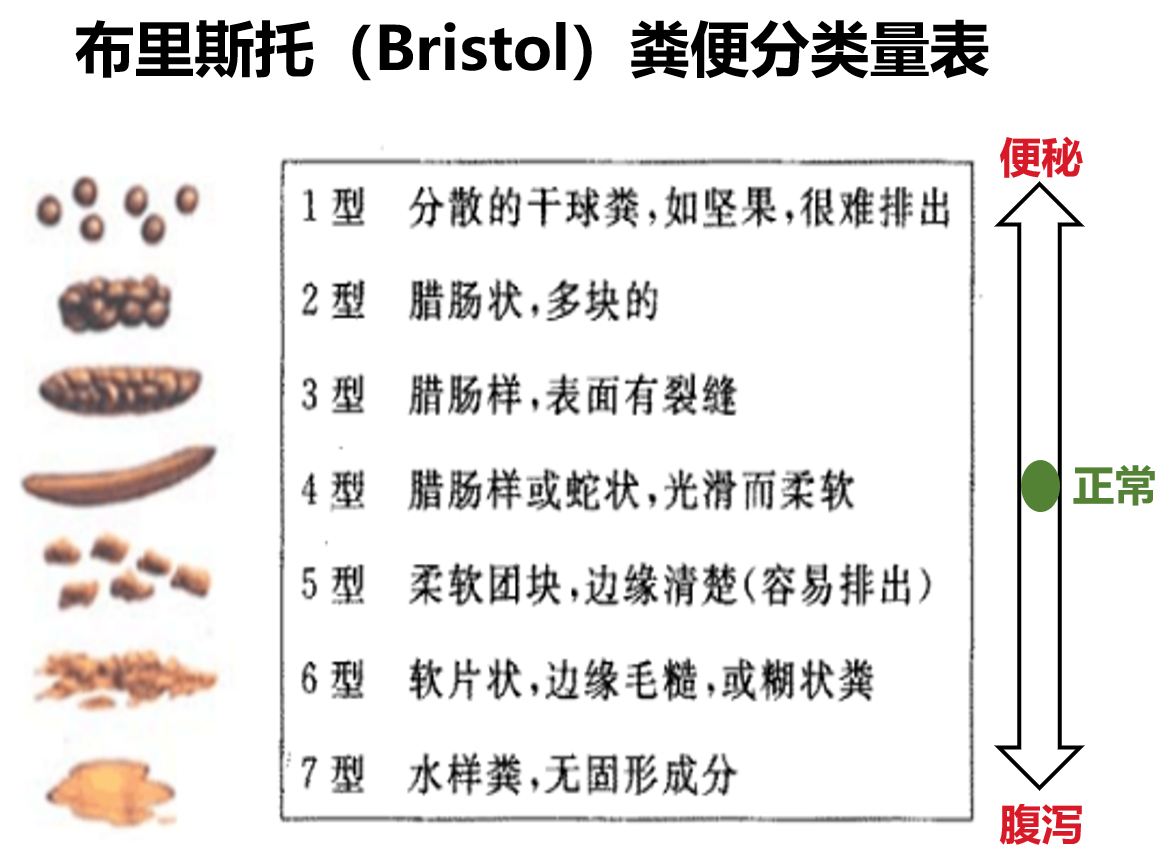


**Type 5: Soft blobs with clear edges (easy to pass)**

**Type 1: Separate hard lumps, like nuts (difficult to pass)**

**Diarrhoea**

**Type 7: Watery; no solid pieces**

**Type 6: Fluffy pieces with ragged edges, or a mushy stool**

**Type 4: Sausage- or snake-like; smooth and soft**

**Type 3: Sausage-like, with cracks on the surface**

**Type 2: Sausage-shaped, lumpy**

**Constipation**

**Normal**

Appendix 3 Bristol Stool Form Scale

(2) Observation period during administration (days 0 to 28):

1) Probiotics group: Volunteers will take *L. plantarum* P9 powder directly or with warm water (below 40°C) on a full stomach, 1 pack (100 billion CFUs) per day; if antibiotics must be taken, probiotics should be taken 2 hours later.

2) Placebo group: Volunteers will take the placebo in the same manner as the probiotics group. The placebo contains no probiotics, comprises maltodextrin (60%), orange powder (20%), and maltitol (20%). and has the same appearance, packaging, and taste as the *L. plantarum* P9 powder.

Both the probiotics and placebo will be stored in a cool, dry place away from direct sunlight.

(3) Postadministration observation period (days 29 to 42): No probiotics or placebo will be taken. All remaining probiotics or placebo that were not taken and empty packages will be collected at the end of this period to monitor compliance.

During each treatment cycle, you must cooperate with your doctor’s evaluation. Your doctor will periodically conduct various evaluations on you in accordance with the regulations of the research plan to evaluate the efficacy and safety of the sample after you receive the treatment.

Additionally, one stool sample for an analysis of the gut microbiome (essential), one stool sample for the metabolomics test (essential) and one whole blood sample for microRNA detection (optional) will be collected from each volunteer on days 0 (preadministration observation period), 28 (observation period during administration) and 42 (postadministration observation period).

**5. Possible benefits of participating in the study**

Your medical condition may be improved by participating in this study. Improvements may be expected in constipation severity, number of bowel movements, and gut flora, but we cannot guarantee that you will benefit from this study.

You will receive probiotics free for 28 days. In addition, each participant will receive a reward of 300 RMB once they complete the follow-up.

**6. Participation in the study/withdrawal from the study/termination of the study**

The decision on whether to participate in the study is entirely up to you. You can refuse to participate in this study, or you can withdraw from this study at any time. Withdrawal/refusal will not affect the relationship between you and the doctor and will not cause the loss of your medical or other benefits.

**7. Alternative treatment options**

In addition to participating in this study, you can choose to receive conventional treatments provided by doctors in the clinic, such as adsorbents and anti-motility agents for treatment. You can discuss other treatments with your doctor.

**8. Confidentiality in the study**

If you agree to participate in this study, your medical records will be reviewed by the executor and supervisor of the study. All your information collected during the research will be kept strictly confidential, and only your contact information will be listed in a form containing identifiable information. We will save the form in a secure database, and we will contact you by phone as needed in the future. However, during data analysis, all your information will be anonymised, and no personal information will be disclosed in future publications or other articles presented to the public.

**9. Risks and discomforts in the study**

After taking probiotics, a gurgling sound in the stomach and slightly more gas production may occur. Some studies have shown that people who are intolerant to taking probiotics might experience constipation, which is considered a "Herxcel reaction". This reaction usually takes a short time to resolve and disappears within a few days to 2 weeks.

Any scientific research has risks, discomforts, and inconveniences, and you should fully consider these factors before agreeing to participate in any clinical trial.

**10. Experimental expense**

During the clinical study period, the sponsor will provide the probiotic product *L. plantarum* P9 and placebo free of charge until the end of the study and provide the examination fees stipulated in the study protocol. During the study period, if serious adverse reactions related to the experimental drugs occur, Jiangzhong Pharmaceutical Co., Ltd. will provide reimbursement for the corresponding treatment costs and economic compensation for damages related in accordance with the relevant laws and regulations of China.

Any other medical conditions that you may have at the same time will not be reimbursed.

**Signature page**

**If you or your family/guardian agree to participate in the study, please read the relevant statement in detail and sign.**

I have been informed of the purpose, method, and possible risks, discomforts, and related benefits of this trial.

I am sure that I have spent sufficient time reading and understanding the aforementioned content, the doctor has explained the medical terms, and I have provided satisfactory answers to all the questions raised by the researchers. I understand that I can voluntarily withdraw from this study at any time without affecting future doctor–patient relationships and treatment. I know that if I have any questions during the trial, I should contact the doctor in charge.

I voluntarily participate in this trial and become a subject of this trial.

Signature of volunteer:

Date of signature:

I have truthfully informed the subject (or designated agent) of the purpose, content, benefits, and possible adverse reactions of this research. I have asked the subject if he or she has any questions about this research and have tried my best to explain the study.

Signature of researcher:

Date of signature:

**Appendix 5 Patient Assessment of Constipation Quality of Life (PAC-QOL) questionnaire**

PAC-QOL

PATIENT ASSESSMENT OF CONSTIPATION

The following questions are designed to measure the effect of constipation on your daily life over the past 2 weeks. For each question, please check one box.

| The following questions ask about **your symptoms** related to constipation. During the past 2 weeks, to what extent or intensity have you？ | Not at all | A little bit | Moderately | Quite a bit | Extremely |
| --- | --- | --- | --- | --- | --- |
|  | **1** | **2** | **3** | **4** | **5** |
| 1. Felt bloated to the point of bursting? | □ | □ | □ | □ | □ |
| 2. Felt heavy because of your constipation? | □ | □ | □ | □ | □ |
| The next few questions ask about how c**onstipation** affects your daily life. During the past 2 weeks, how much of the time have you？ | None of the  time | A little of  the time | Some of the  time | Most of the  time | All of the  time |
|  | **1** | **2** | **3** | **4** | **5** |
| 3. Felt any physical discomfort? | □ | □ | □ | □ | □ |
| 4. Felt the need to have a bowel movement but not been able? | □ | □ | □ | □ | □ |
| 5. Been embarrassed to be with other people? | □ | □ | □ | □ | □ |
| 6. Been eating less and less because of not being able to have bowel movements? | □ | □ | □ | □ | □ |
| The next few questions ask about how **constipation affects** your daily life. During the past 2 weeks, to what extent or intensity have you... | Not at all | A little bit | Moderately | Quite a bit | Extremely |
|  | **1** | **2** | **3** | **4** | **5** |
| 7. Had to be careful about what you eat? | □ | □ | □ | □ | □ |
| 8. Had a decreased appetite? | □ | □ | □ | □ | □ |
| 9. Been worried about not being able to choose what  you eat (for example, at a friend’s house)? | □ | □ | □ | □ | □ |
| 10. Been embarrassed about staying in the bathroom  for so long when you were away from home? | □ | □ | □ | □ | □ |
| 11. Been embarrassed about having to go to the  bathroom so often when you were away from home? | □ | □ | □ | □ | □ |
| 12. Been worried about having to change your daily  routine (for example, travelling and being away from home)? | □ | □ | □ | □ | □ |
| The next few questions ask about **your feelings** related to constipation. During the past 2 weeks, how much of the time have you... | None of the  time | A little of  the time | Some of the  time | Most of the  time | All of the  time |
|  | **1** | **2** | **3** | **4** | **5** |
| 13. Felt irritable because of your condition? | □ | □ | □ | □ | □ |
| 14. Been upset by your condition? | □ | □ | □ | □ | □ |
| 15. Felt obsessed by your condition? | □ | □ | □ | □ | □ |
| 16. Felt stressed by your condition? | □ | □ | □ | □ | □ |
| 17. Felt less self-confident because of your condition? | □ | □ | □ | □ | □ |
| 18. Felt in control of your situation? | □ | □ | □ | □ | □ |
| The next questions ask about **your feelings** related to constipation. During the past 2 weeks, to what extent or intensity have you... | Not at all | A little bit | Moderately | Quite a bit | Extremely |
|  | **1** | **2** | **3** | **4** | **5** |
| 19. Been worried about not knowing when you are  going to be able to have a bowel movement? | □ | □ | □ | □ | □ |
| 20. Been worried about not being able to have a bowel  movement? | □ | □ | □ | □ | □ |
| 21. Been increasingly bothered by not being able to have  a bowel movement? | □ | □ | □ | □ | □ |
| The next questions ask about **your life** with constipation. During the past 2 weeks, how much of the time have you... | None of the  time | A little of  the time | Some of the  time | Most of the  time | All of the  time |
|  | **1** | **2** | **3** | **4** | **5** |
| 22. Been worried that your condition will get worse? | □ | □ | □ | □ | □ |
| 23. Felt that your body was not working properly? | □ | □ | □ | □ | □ |
| 24. Had fewer bowel movements than you would like? | □ | □ | □ | □ | □ |
| The next questions ask about your **degree of satisfaction related** to constipation. During the past 2 weeks, to what extent or intensity have you been... | Not at all | A little bit | Moderately | Quite a bit | Extremely |
|  | **1** | **2** | **3** | **4** | **5** |
| 25. Satisfied with how often you have a bowel movement? | □ | □ | □ | □ | □ |
| 26. Satisfied with the regularity of your bowel movements? | □ | □ | □ | □ | □ |
| 27. Satisfied with the time it takes for food to pass through  the intestines? | □ | □ | □ | □ | □ |
| 28. Satisfied with your treatment? | □ | □ | □ | □ | □ |

**Appendix 6 Depression, Anxiety and Stress Questionnaire**

**Depression Anxiety Stress Scale 21**

Please read each statement and select the response that best describes how much the statement applied to you over the past week. Please try to answer every question. Answers are not right or wrong.

The rating scale is as follows:

0 — Did not apply to me at all

1 — Applied to me some degree, or some of the time

2 — Applied to me a considerable degree, or a good part of time

3 — Applied to me very much, or most of the time

| 1.  I found it hard to wind down. | 0 | 1 | 2 | 3 |
| --- | --- | --- | --- | --- |
| 2.  I was aware of dryness in my mouth. | 0 | 1 | 2 | 3 |
| 3.  I couldn’t seem to experience any positive feeling at all. | 0 | 1 | 2 | 3 |
| 4.  I experienced breathing difficulty. | 0 | 1 | 2 | 3 |
| 5.  I found it difficult to work up the initiative to do things. | 0 | 1 | 2 | 3 |
| 6.  I tended to overreact to situations. | 0 | 1 | 2 | 3 |
| 7.  I experienced trembling (e.g., in the hands). | 0 | 1 | 2 | 3 |
| 8.  I felt that I was using a lot of nervous energy. | 0 | 1 | 2 | 3 |
| 9.  I was worried about situations in which I might panic and make a fool of myself. | 0 | 1 | 2 | 3 |
| 10.  I felt that I had nothing to look forward to. | 0 | 1 | 2 | 3 |
| 11.  I found myself getting agitated | 0 | 1 | 2 | 3 |
| 12.  I found it difficult to relax. | 0 | 1 | 2 | 3 |
| 13.  I felt downhearted and blue. | 0 | 1 | 2 | 3 |
| 14.  I was intolerant of anything that kept me from completing what I was doing. | 0 | 1 | 2 | 3 |
| 15.  I felt I was close to panic. | 0 | 1 | 2 | 3 |
| 16.  I was unable to become enthusiastic about anything. | 0 | 1 | 2 | 3 |
| 17.  I felt that I wasn’t worth much as a person. | 0 | 1 | 2 | 3 |
| 18.  I felt I was rather touchy. | 0 | 1 | 2 | 3 |
| 19. I was aware of the action of my heart in the absence of physical exertion. | 0 | 1 | 2 | 3 |
| 20.  I felt scared without any good reason. | 0 | 1 | 2 | 3 |
| 21.  I felt that life was meaningless. | 0 | 1 | 2 | 3 |

| **Appendix 7 The comparisons of methodologies among the present and previous RCTs related to the effect of probiotics on chronic constipation.** | | | | | | | | | | | | |
| --- | --- | --- | --- | --- | --- | --- | --- | --- | --- | --- | --- | --- |
| **Study** | **Design** | **Allocation concealment/Blinding/ITT analysis/Description of withdrawals or dropouts** | **Sample size (probiotics:control)** | **Population** | **Age (years)** | **Diagnostic Criteria** | **Intervention** | **Comparator** | **Probiotic strain** | **Outcomes** | **Daily dosage (CFUs)** | **Study stage (duration)** |
| Agrawal, 2009 [68] | Randomized, double-blind, controlled, parallel group study | Yes/Yes/Yes/Yes | 17/17 | Irritable bowel syndrome with constipation in females | 20-69 | Rome III criteria for constipation predominant IBS | A fermented milk containing probiotics | A milk-based nonfermented dairy product | *Bifidobacterium lactis* | Abdominal distension, gastrointestinal transit, abdominal symptoms and bowel habit | 1.25x10^10^ | Screening period/baseline (11 days), administration period (28 days), postadministration period (7 days) |
| Holma, 2010 [64] | Randomized, controlled, unblinded, 2×2 factorial design | Unclear/Unclear/No/No | 10/12/11/10/8^a^ | Adults with self-reported constipation | 22-78 | <5 defecations/week without laxatives or <7 defecations/week with laxatives, and self-reported constipation | Whole-grain rye bread or *Lactobacillus rhamnosus* GG (LGG) or whole-grain rye bread+LGG | Laxatives or white wheat bread | Cultured buttermilk supplemented with *Lactobacillus* GG | Faecal weight, pH, short-chain fatty acids (SCFA) and bacterial enzyme activities, total intestinal transit time (TITT), and breath hydrogen | 2x10^10^ | Baseline period (1 wk)-intervention period (3 wks) |
| Hongisto, 2006 [69] | Randomized, controlled, 2×2 factorial design | No/No/No/No | 14/15/16/14^b^ | Women with self-reported constipation | 18-57 | Feelings of reduced/less-frequent bowel movements, as well as straining at defecation | Fibre-rich rye bread + *Lactobacillus rhamnosus* GG (LGG) | Fibre-rich rye bread, LGG or low-fibre toast | *Lactobacillus* GG | TITT, faecal frequency and consistency, difficulty in defecation and gastrointestinal symptoms | 1.5x10^10^ | Screening/baseline period (1 wk)- administration period (3 wks)- postadministration period (3 wks) |
| Malpeli, 2012 [70] | Randomized, double-blind, placebo-controlled and crossover | Yes/Yes/No/Yes | 28/35 | healthy women | 21-60 | Those with a slow transit perception and/or abdominal pain (bloating) or slow transit (functional constipation) according to Rome III criteria | The synbiotic yoghurt contained the test probiotics | The standard yoghurt | *Bifidobacterium infantis* and *Lactobacillus casei* | Intestinal transit time, voiding frequency, stool consistency and bloating, intestinal flora | 10^9^-10^10^ | Screening/baseline period (15 days)-administration period (15 days)-washout period (15 days)-administration period (15 days) |
| Waller, 2011 [71] | Sex-stratified, triple-blind, placebo-controlled, parallel-group, dose-ranging study | Unclear/Yes/No/Yes | 33/33/34^c^ | adults with constipation | 25-65 | Self-report of stool type 2–4 on the Bristol Stool Chart and an average of 1–3 bowel movements per week. | The capsules contained the test probiotics | placebo | *Bifidobacterium lactis* | Food frequency, whole gut transit time, functional gastrointestinal symptom frequency | 17.2×10^9^ or 1.8×10^9^ | Screening/baseline period (7 days)-administration period (14 days) |
| Krammer, 2011 [72] | Randomized double-blind placebo-controlled trial | Unclear/Yes/No/No | 12/12 | female adults with chronic constipation | Unclear (~50) | Transit time >72 h. | Ferment­ed milk drink containing test probiotics | placebo | *Lactobacillus casei* Shirota (LcS) | Colonic transit time, stool frequency and consistency, constipation-related and gastrointestinal symptoms | 6.5×10^9^ | Screening/baseline period (2 wks)-administration period (4 wks)- postadministration period (4 wks) |
| Bazzocchi, 2014 [73] | Randomized double-blind, controlled trial | Yes/Yes/No/No | 17/12 | patients with severe functional constipation | 19–65 | Constipation consecutively matching the Rome III Diagnostic Criteria for functional constipation | A synergic mixture of the prebiotic psyllium fibre and five probiotic strains | maltodextrin | *L. plantarum*, *L. acidophilus* and *L. rhamnosus* and *B. longum spp. longum* and *B. breve* species | % Bowel motions with normal stools, decrease in Agachan–Wexner score for constipation severity, increase in faecal levels of *Lactobacillus* and *Bifidobacterium* | unclear | Screening/baseline period (2 wks)- administration period (8 wks) |
| Cudmore, 2017 [74] | Randomized, double-blind, placebo-controlled clinical study | Yes/Yes/Yes/Yes | 35/34 | adults with chronic, functional constipation | 18-80 | Rome III diagnostic criteria for functional constipation | 5 g sachet containing test probiotics | placebo | *L. rhamnosus*, *B. bifidum*, *L. acidophilus*, *L. plantarum*, *Lactobacillus bulgaricus* | Number of bowel movements, stool consistency, quality of life, constipation symptoms, reduced laxative use by the subjects | 1.2x10^9^ | Screening/baseline period (14 d)- administration period (7 d) |
| Dorte Eskesen, 2015 [65] | Randomized, double-blind, placebo-controlled | Yes/Yes/Yes/No | 343/452/453^d^ | healthy subjects with constipation | 18-70 | Low defecation frequency (2–4 times/week) and complaints of general abdominal discomfort | Probiotic strain in capsule form | placebo | *Bifidobacterium animalis* subsp. *Lactis* | Defecation frequency and gastrointestinal well-being responder rates, symptom severity scores for abdominal pain and bloating | 1×10^9^ or 10×10^9^ | Screening/baseline period (2 wks)- administration period (4 wks) |
| Mena Mustapha Mazlyn, 2013 [75] | Randomized, double-blind, placebo-controlled | Unclear/Yes/yes/Yes | 47/43 | adults with functional constipation | 18-60 | Rome II criteria | Shirota fermented milk containing the test probiotics | placebo | *Lactobacillus casei* strain Shirota | Constipation severity, stool frequency, stool consistency and quantity | 3.0 × 10^10^ | Screening/baseline period (2 wks)- administration period (4 wks) |
| Veronica Ojetti, 2014 [76] | Randomized, double-blind, placebo-controlled | Unclear/Yes/yes/Yes | 20/20 | adults with functional constipation | Unclear (approximately 36±15) | Rome III | Probiotic tablets containing the test probiotics | placebo | *Lactobacillus reuteri* | Bowel movements/week frequency, stool consistency according to BSS | 2×10^8^ | Administration period (4 wks) |
| Yoshiyuki TANAKA, 2015 [77] | Randomized, double-blind, placebo-controlled | Unclear/Yes/Unclear/Yes | 18/20 | adults with constipation | 25-59 | Frequency of bowel movements of <5.0 times/week assessed using a questionnaire | Milk-like drink containing test probiotics | placebo | *B. animalis* subsp. *lactis* | Intestinal *Bifidobacteria*, frequency of defecation | 1.5×10^10^ | Screening/baseline period (2 wks)-administration period (8 wks) |
| Dan L. Waitzberg, 2013 [78] | Randomized, double-blind, placebo-controlled | Unclear/Yes/Unclear/Yes | 50/50 | constipated adult women | 18-75 | Rome III | Synbiotic containing multiple probiotics | placebo | *L. paracasei*, *L. rhamnosus*, *L. acidophilus*, *B. lactis* | Stool frequency, consistency and shape, abdominal pain, bloating and flatulence, constipation intensity | 10^8^-10^9^ | Screening/baseline period (1 wk)- administration period (30 days) |
| Yue-Xin Yang, 2008 [79] | Randomized,  placebo-controlled | Unclear/Unclear/Yes/No | 59/56 | adult females with constipation | 25-65 | Less than three stools per week, increased stool hardness, nonorganic constipation and habitual constipation | Fermented milk contains probiotics | acidified milk | *B. lactis* | Stool frequency, defecation condition scores, stool consistency and food intake, safety evaluation | 1.25×10^10^ | Screening/baseline period (1 wk)- administration period (2 wks) |
| Corinna Koebnick PhD, 2003 [80] | Randomized, double-blind, placebo-controlled | Unclear/Yes/Unclear/No | 35/35 | adults with chronic idiopathic constipation | 18-70 | NA | Probiotic beverage | placebo | *L. casei* strain Shirota | Severity of constipation, defecation frequency, stool consistency, occurrence and degree of flatulence, occurrence, and degree of bloating | 6.5×10^9^ | Screening/baseline period (2 wks)- administration period (4 wks) |
| LING-NAN BU, 2007 [81] | Randomized, double-blind, placebo-controlled | Yes/Yes/Yes/Yes | 18/18/9^e^ | children with chronic constipation | <10 | Stool frequency of <3 times per week for >2 months and at least one of the following minor criteria: anal fissures with bleeding due to constipation, faecal soiling, or passage of large and hard stool. | Capsules containing probiotics | magnesium oxide (traditional laxative) or placebo | *L. casei rhamnosus* | Frequency of daily bowel movements, stool consistency, abdominal pain, faecal soiling, intestinal flora | 8 × 10^8^ | Administration period (4 wks) |
| ALEKSANDRA BANASZKIEWICZ, 2005 [82] | Randomized, double-blind, placebo-controlled | Yes/Yes/Yes/No | 43/41 | children with constipation | 2-16 | <3 spontaneous bowel movements per week for at least 12 weeks | Lactulose with *Lactobacillus* GG | lactulose with placebo | *L. rhamnosus* GG | Greater than or equal to 3 spontaneous BMs per week with no episodes of faecal soiling, the number of BMs per week, number of episodes of faecal soiling per week, stool consistency, and straining frequency per week, percentage of patients using laxatives was assessed at 24 weeks | 2×10^9^ | Administration period (12 wks)-postadministration period |
| BOUVIER, 2001 [83] | Double blind, placebo-controlled parallel study | Yes/Yes/No/No | 36/36 | healthy adults | 21-42 | Normally indicated by medical examination and not taking any medication for at least four weeks | Fermented milk containing the test probiotics | placebo milk | *B. animalis* | Colonic transit time | 9.75×10^10^ | Screening/baseline period (10 days)- administration period (11 days) |
| MARTEAU, 2002 [84] | Double-blind, randomized, controlled study | Unclear/Yes/No/No | 17/15 | healthy women | 18-45 | Judged by a medical examination | Fermented milk containing the probiotics | fermented milk | *B. animalis* strain | Total and sigmoid transit times, the other transit times, faecal weight, pH, bacterial mass, and bile acids | 0.19-1.9×10^10^ | Screening/baseline period (10 days)- administration period (10 days)-washout period (10 days)-administration period (10 day) |
| Merenstein, 2014 [85] | Triple-blind, placebo-controlled, two-period crossover trial | Yes/Yes/Yes/Yes | 34/34 | women | 18-65 | Self-reported history of straining during bowel movements or hard or lumpy stools in the past 2 years | Yogurt containing the test probiotics | yogurt | *B. animalis ssp. lactis* | Colonic transit time, the number of bowel movements/week, QOL, frequency of bowel movements over 2 weeks, frequency of constipated stools, % positive for *B. animalis ssp. Lactis*, daily diet, compliance | 2.0-5.6×  10^10^ | Screening/baseline period (duration is unclear)-administration period (14 days)-washout period (6 wks)-administration period (14 days) |
| Akihiro Ishizuka, 2012 [86] | Placebo-controlled double-blind, crossover | Unclear/Yes/Yes/Yes/No | 12/12 | adults suffering from constipation | 20-23 | Number of defecations is less than or equal to 5.0 times/week | Milk-like drink containing the test probiotics | milk-like drink | *B. lactis* | Intestinal *Bifidobacteria* | 1×10^10^ | Screening/baseline period (2 wks)-administration period (2 wks)-washout period (2 wks)-administration period (2 wks) |
| Mario Del Piano, 2010 [87] | Randomized, double-blind, placebo-controlled study | Unclear/Yes/Yes/Yes/Yes | 80/110/110^f^ | healthy volunteers with evacuation disorders and hard stools | 24-71 | Judged by a complete physical examination, normal values of laboratory tests, and no evidence of gastrointestinal disease on plain abdominal X-ray and ultrasound | A half glass of water containing the test probiotics | placebo | Mixture of *L. plantarum* and *B. breve* or *B. animalis* subspecies *lactis* | Ease of expulsion, number of weekly evacuations, anal itching, burning, and pain abdominal bloating, sensation of complete emptying | 5 × 10^9^ or 2.5 × 10^9^ | Screening/baseline period (7 days)-observation period during administration (30 days) |
| G. Riezzo, 2018 [88] | Randomized, double-blind, placebo-controlled | Unclear/Yes/No/Yes | 28/28 | adults with functional constipation | 19-65 | Rome III criteria for FC without matching Rome criteria for IBS | *L. reuteri* DSM 17938 | placebo | *L. reuteri* | CSS, PAC-QoL | 2×10^8^ and 4×10^8^ | Screening/baseline period (7 days)-observation period 1 during administration (15 days with the high dosage)-observation period 2 during administration (90 days with the low dosage) |
| G. Riezzo, 2012 [89] | Randomized, double-blind, crossover study | Unclear/Yes/Yes/Yes | 10/10 | adults with functional constipation | 19-70 | Rome Criteria III for constipation 20, Constipation Scoring System (CSS) | Probiotic-enriched artichokes | artichokes | *L. paracasei* | Stool consistency, GSRS sum score, SCFAs, colonic transit time | 2×10^10^ | Screening/baseline period (7 days)-observation period during administration (15 days)-washout period (4 wk)-observation period during administration (15 days) |
| Eirini Dimidi, 2019 [46] | Randomized, double-blind, placebo-controlled | Unclear/Yes/Yes/Yes | 37/38 | general population with mild constipation | 18-65 | Modified Rome III diagnostic criteria for functional constipation | Milk powder with probiotics | placebo milk powder | *B. lactis* | Whole gut transit time, regional gut transit time, constipation severity, stool frequency and stool consistency, QOL, gut microbiota composition, safety outcomes | 1.5 x 10^10^ | Screening/baseline period (duration is unclear)-observation period during administration (4 wks)-postadministration observation period (8 wks) |
| Mojgan Mirghafourvand, 2016 [90] | Randomized, triple-blind, placebo-controlled | Unclear/Yes/No/Yes | 29/28 | constipated pregnant women with a gestational age of 24-28 weeks | >18 | Rome III criteria | Probiotic yogurt containing the test probiotics | conventional yogurts | *L. acidophilus* and *B. lactis* | The defecation frequency, stool consistency, straining during defecation, sensation of anorectal obstruction, sensation of incomplete evacuation and manual manoeuvres to facilitate defecation, the amount of defecation, stool colour, and QOL | 4.8×10^8^ | Screening/baseline period (duration is unclear)-observation period during administration (4 wks)-postadministration observation period (2 wks) |
| Favretto, 2013 [66] | Randomized controlled trial | Unclear/No/No/No | 15/15 | constipated women | 20-60 | Rome III consensus | Fresh cheese containing the test probiotics | regular fresh cheese | *B. lactis* | Symptoms of Rome III criteria | 1×10^8^ | Screening/baseline period (duration is unclear)-observation period during administration (30 d) |
| De Paula, 2008 [91] | Open, randomized, controlled study in parallel groups with intercrossing | Yes/No/No/Yes | Varied according to different outcomes | women with functional constipation (266) and women without constipation (112) | 18-55 | Rome II criteria | Dessert with probiotics | lacteous dessert | *B. animalis* | Stool frequency> 5/week, stool frequency > 3/week, stool shape, straining effort and pain during bowel evacuation, stool frequency (bowel movements/week), stool shape, pain and straining effort associated with bowel evacuation | 1×10^8^ | Screening/baseline period (duration is unclear)-observation period during administration (2 wks)-observation period during cross-administration (2 wks) |
| Ding, 2016 [92] | Prospective Randomized Trial | Yes/Yes/No/Yes | 48/45 | patients with slow transit constipation | <18 | Rome III criteria for chronic constipation | Synbiotic containing the test probiotics | placebo | Unclear | Stool frequency and consistency, colonic transit time (CTT), evacuation and abdominal symptoms, patient assessment of constipation symptoms, gastrointestinal QOL index scores, satisfaction scores, and adverse events | unclear | Screening/baseline period (duration is unclear)-observation period during administration (12 wks) |
| Fateh, 2011 [93] | Double-blind, randomized, placebo-controlled trial | Unclear/Yes/No/no | 31/29 | young men suffering from functional constipation | >18 | Rome III criteria for chronic constipation | Synbiotic mixture of probiotics | capsules | *L. casei*, *L. rhamnosus*, *Streptococcus thermophilus*, *B. breve*, *L. acidophilus*, *B. longum*, *L. bulgaricus* | Stool frequency per week, overall score of patient assessment of constipation symptoms, the Bristol stool form scale, abdominal symptoms score, rectal symptoms score, stool symptoms score | 1×10^8^ | Screening/baseline period (duration is unclear)-observation period during administration (4 wks) |
| Jayasimhan, 2013 [94] | Randomized, double-blind, placebo-controlled | Yes/Yes/Yes/Yes | 50/58 | constipated adults | 18-81 | Rome III criteria for chronic constipation | Microbial cell preparation containing probiotics | placebo | *L. acidophilus*, *L. casei*, *L. lactis*, *B. bifidum*, *B. longum* and *B. infantis* | Frequency of bowel movements per week, self-perception of the improvement in symptoms (straining, lumpy or hard stool, sensation of incomplete evacuation, sensation of anorectal blockage and manual manoeuvres to aid in defecation) | 3×10^10^ | Screening/baseline period (duration is unclear)-observation period during administration (1 wk) |
| Magro, 2014 [95] | Randomized, double-blind, controlled study | No/Yes/No/Yes | 21/26 | individuals with chronic constipation | 18-45 | Rome III criteria for chronic constipation | Yogurt containing probiotics | yogurts | *L. acidophilus* and *B. lactis* | Agachan’s score, bowel movements/day, colonic transit time (hours) | >1×10^9^ | Screening/baseline period (duration is unclear)-observation period during administration (2 wks) |
| Kondo, 2013 [67] | Double-blind, placebo-controlled, parallel-group design | Yes/Yes/No/Yes | 32/34 | elderly patients with constipation | >65 | Unclear | Powder containing probiotics | placebo powder | *B. longum* | Times of defecation, stool form and consistency | 2.5×10^10^ | Screening/baseline period (1 wk)-observation period during administration (16 wks) |
|  |  |  | 32/37/33^g^ | elderly patients with constipation | >65 | Unclear | Powder containing probiotics | powder | *B. longum* | Bowel movements, faecal microbiota, stool form and consistency | 2.5 × 10^10^ or 5×10^10^ | Screening/baseline period (1 wk)-observation period during administration (16 wks) |
| Yoon, 2018 [96] | Randomized, double‑blind, placebo‑controlled Study | No/Yes/No/Yes | 90/90 | adults with chronic constipation | 18-75 | Rome IV criteria | Chocolate case containing probiotics | placebo | *S. thermophilus* and *L. plantarum* | Faecal microbiota, global improvement scale, frequency of bowel movement, Bristol Stool Form Scale and Complete Spontaneous Bowel Movements (CSBM), Gastrointestinal Symptom Rating Scale, health-related QOL | 3.0 × 10^8^ and 1.0 × 10^8^ | Screening/baseline period (1 wk)-observation period during administration (4 wks)-postadministration observation period (4 wks) |
| The present study | Randomized, double-blind, placebo-controlled clinical trial | Yes/Yes/Yes/Yes | 100/100 | adults with chronic constipation | 18-65 | Rome IV criteria | Powder containing probiotics | placebo | *L. plantarum* P9 | Frequency of CSBMs per week, weekly mean frequency of CSBMs >3, weekly mean frequency of SBMs, weekly mean stool appearance score, weekly mean difficulty of passing stool score, WPUAMA, QOL score, emotional status score, gut microbiome, and faecal metabolome | >1×10^11^ | Screening/baseline period (2 wks)-observation period during administration (4 wks)-postadministration observation period (2 wks) |

Notes: The samples sizes correspond to the following groups: a, groups receiving rye bread, LGG, rye bread+LGG, control, and laxative, respectively; b, the groups receiving rye bread+LGG, rye bread, LGG, and control, respectively; c, groups receiving the high dosage, low dosage, and placebo, respectively; d, groups receiving the low dosage, high dosage, and placebo, respectively; e, groups receiving MgO, probiotics, placebo, respectively; f, groups receiving placebo, probiotics1, and probiotics2, respectively; and g, groups receiving the low dosage, high dosage, and control, respectively.

**Appendix 8 Copy of National Natural Science Foundation of China with English translation**


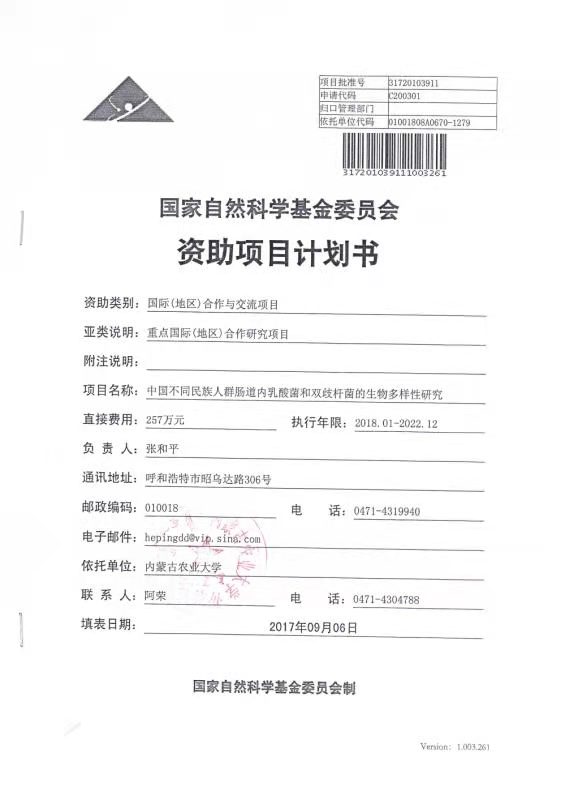


Project approval number: 31720103911

Application code: C200301

Central Management Department:

Relying unit code: 01001808A0670-1279

National Natural Science Foundation of China

Funding Project Plan

Funding category: International (regional) cooperation and exchange projects

Subcategory description: Key international (regional) cooperative research projects

Note description:

Project name: Study on Biodiversity of *Lactobacillus* and *Bifidobacterium* in the Intestines of Different Ethnic Groups in China

Direct cost: ¥2.57 million

Execution period: January 2018 to December 2022

Person in charge: Heping Zhang

Mailing address: No. 306 Zhaowuda Road, Hohhot

Postal Code: 010018 Phone: 0471-439940

E-mail: hepingdd@vip.sina.com

Supporting unit: Inner Mongolia Agricultural University

Contact: A Rong Phone: 0471-4304788

Date of completion: September 06, 2017

National Natural Science Foundation of China
